# A Reciprocal Interaction between β-Catenin and Osterix in Cementogenesis

**DOI:** 10.1038/s41598-017-08607-5

**Published:** 2017-08-15

**Authors:** Hwajung Choi, Tak-Heun Kim, Siqin Yang, Jeong-Chae Lee, Hyung-Keun You, Eui-Sic Cho

**Affiliations:** 1 Cluster for Craniofacial Development and Regeneration Research, Institute of Oral Biosciences, Chonbuk National University School of Dentistry, Jeonju, 54896 South Korea; 2 Department of Periodontology, School of Dentistry, Wonkwang University, Iksan, 54538 South Korea

## Abstract

Although accumulating evidence indicates that both β-catenin and osterix (Osx) are essential for bone and tooth development, few studies have investigated the interaction of these two key proteins in the context of cementogenesis. In this study, we used transgenic mice with constitutively active *β-catenin* and inactive *Osx* in the dental mesenchyme to address this question. We found that cementoblasts with constitutively active β-catenin require Osx to produce excessive cellular cementum, and that ablation of *Osx* prevents this abnormal accumulation. Importantly, cementoblasts transduced with retrovirus expressing constitutively active β-catenin exhibited upregulation of Osx expression through direct binding to the promoter region of *Osx*. Osx regulates Lef1 expression and consequently could regulate T-cell factor/lymphoid enhancer factor (Tcf/Lef) binding activity in Wnt/β-catenin signaling. However, the loss of Tcf/Lef binding activity by *Osx* ablation was not rescued by transduction of retrovirus expressing constitutively active β-catenin or ectopic Lef1 overexpression. These results suggest that the Tcf/Lef binding activity of Wnt/β-catenin signaling is Osx-dependent during cementogenesis. Moreover, Osx differentially regulates the expression of various Tcf family members, suggesting that Osx regulates cementogenesis by utilizing various Tcf/Lef-dependent mechanisms. This is the first report to show that downstream Osx signaling through Tcf/Lefs is critical for cementogenesis.

## Introduction

Cementum is a thin mineralized tissue covering the tooth root surface. Since cementum helps anchor the tooth to the surrounding alveolar bone and also provides a mechanical barrier to root resorption, this tissue is important for maintaining periodontal homeostasis and for resisting the continuous occlusal force^1, 2^. Although cementum has many similarities to bone, particularly in its biochemical composition and biomechanical properties, unlike bone, cementum is resistant to remodeling in its physiological state and is hard to regenerate.

Wnt/β-catenin signaling plays multiple roles in various stages of tooth morphogenesis^3, 4^. Although its essential roles in tooth morphogenesis have been well studied, little is known about the involvement of Wnt/β-catenin signaling in cellular differentiation and dental hard tissue formation. The transcriptional activity of β-catenin is tightly controlled by targeting a protein complex consisting of Adenomatous polyposis coli (Apc), the scaffolding protein Axin, casein kinase 1 (CK-1), and glycogen synthase kinase 3β (Gsk-3β) for its proteasomal degradation^5, 6^. After stabilization and nuclear accumulation, β-catenin engages T-cell factor/lymphoid enhancer factor (Tcf/Lef) transcription factors to activate the transcriptional program in the nucleus^7^. The Tcf family includes Lef1, Tcf1, Tcf3, and Tcf4; Tcf/Lefs are intensively studied as nuclear effectors of Wnt/β-catenin signaling^8, 9^. These effectors cooperate with other factors to regulate Wnt-independent transcription as well as to mediate or suppress Wnt signaling^10, 11^. Moreover, there is increasing evidence of functional diversity and non-redundant activities among Tcf family members^8, 9, 12^. Although Tcf/Lefs are context-dependent regulators of Wnt/β-catenin, their functional implications on cementogenesis and Osx-mediated regulation are largely unknown.

We previously reported excessive cementum formation in *OC-Cre:Catnb* ^*lox/*+^ (*OC-Catnb*) transgenic mice^13^, in which stabilization of β-catenin is constitutively induced. This stabilization is achieved by the elimination of the entire exon 3 sequence, which encodes Gsk-3β phosphorylation targets in osteocalcin (OC)-expressing dental mesenchyme^14^. The excess cementum containing cementocyte-like cells within its matrix was deposited over the cervical and apical regions of the molar root surface. Histological studies of this excess cementum supported the proposal that stabilized β-catenin induces cementoblast differentiation and boosts matrix secretion; however, the precise molecular mechanisms of these processes remain unknown.

Osterix (Osx), a zinc finger-containing transcription factor, was initially identified as a key regulator of osteoblast differentiation during bone formation^15^. We recently showed that Osx is required for overall tooth root formation by regulating odontoblast differentiation, maturation, and root elongation in a site-specific manner^16^. In addition, a genetic study reported that Osx has a vital function in cementogenesis, particularly in cementoblast differentiation. This conclusion was supported by the finding that cementogenesis is impaired following conditional disruption of *Osx* in the dental mesenchyme and is restored by overexpression of *Osx* ^17^. Studies of transgenic animals with stabilized *β-catenin* and transgenic animals overexpressing *Osx* found similar anabolic responses in cementum formation in both animal lines^13, 17^, implying a linked molecular signaling cascade mediated by β-catenin and Osx.

In this study, using *in vivo* and *in vitro* approaches, we demonstrated a reciprocal interaction between β-catenin and Osx in cementogenesis and uncovered the underlying molecular mechanisms. Specifically, we showed that Wnt/β-catenin signaling regulates Osx expression for cementoblast differentiation and cementum matrix secretion; Osx, in turn, regulates Wnt/β-catenin activity by controlling Tcf/Lef expression. This is the first demonstration that the relationship between β-catenin and Osx is critical for cementum formation during postnatal tooth development. These results provide important insight into the mechanisms of cementum formation in tooth development and suggest future strategies for promoting cementum regeneration in periodontal disease.

## Results

### *In vivo* regulation of cementum formation by stabilized β-catenin and Osx

**Figure 1:**
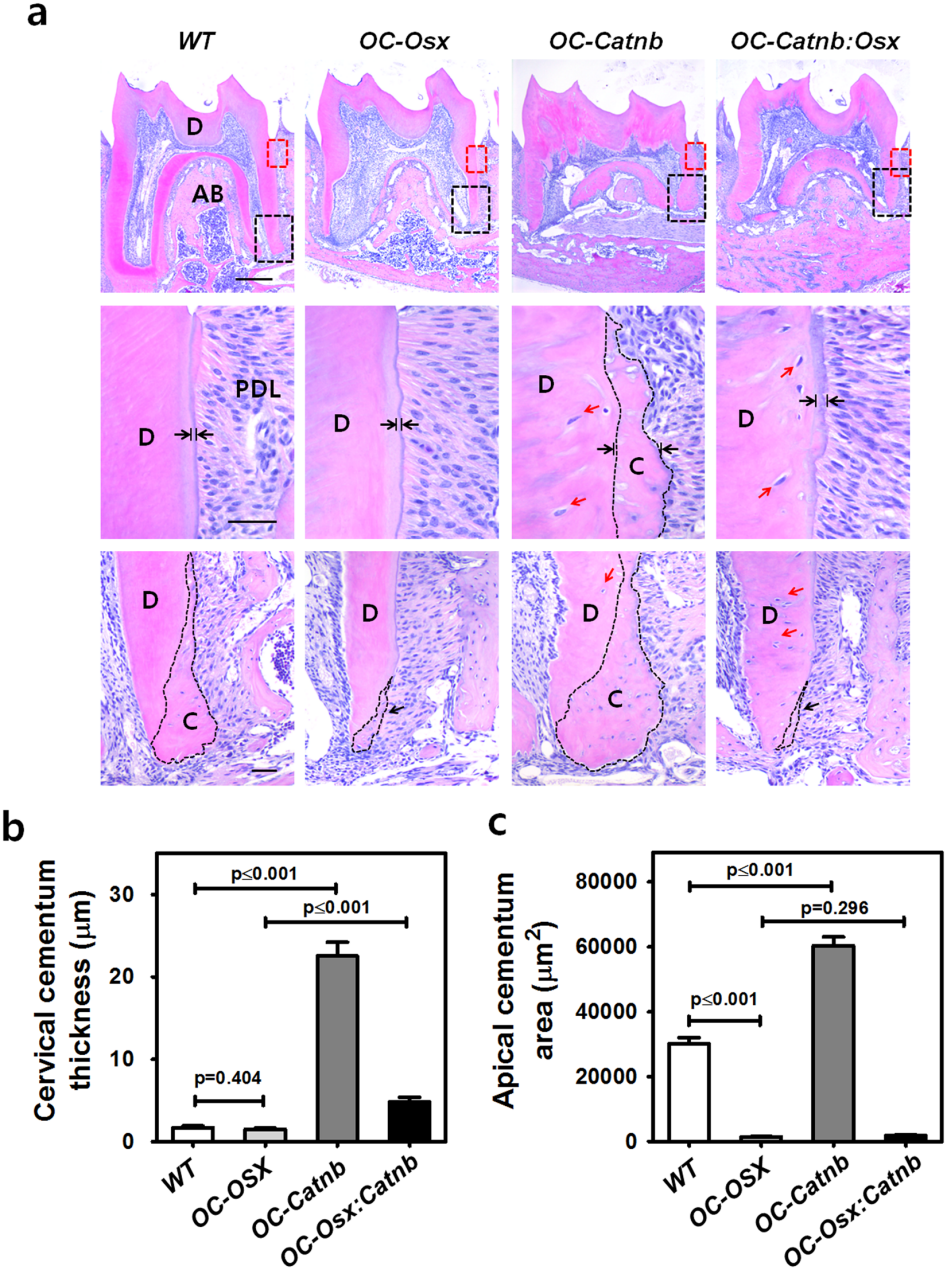
*In vivo* regulation of cementum formation by stabilized β-catenin and Osx. (**a**) Hematoxylin and eosin (H-E)-stained whole sections of mandibular first molars from WT, *OC-Osx*, *OC-Catnb*, and *OC-Catnb:Osx* mice at postnatal week 6 (P6W) (Top). Cervical cementum regions of the red dotted square are shown in the high-powered field (middle row, **a**). The average cervical cementum thickness is indicated by a pair of black arrows in each group. Apical cellular cementum regions of the black dotted square were also magnified (bottom row, **a**). Reduced apical cellular cementum is indicated by black arrows in *OC-Osx* and *OC-Catnb:Osx*, respectively. Red arrows indicate odontoblasts entrapped in osteodentin, which is specifically formed by stabilized β-catenin in OC-expressing odontoblasts. This phenomenon was only observed in *OC-Catnb* and *OC-Catnb:Osx* mice. Scale bars: 400 μm (top), 20 μm (middle), 50 μm (bottom). D, dentin; AB, alveolar bone; PDL, periodontal ligament; C, cementum. Thickness of the cervical cementum (**b**) and area of the apical cellular cementum (**c**) in each group. Values were measured with the distal root of the mandibular first molar. Data represent the mean ± SD of three measurements of five representative slides from each group. Significance was assigned for p-values as indicated.

We previously reported excessive cementum formation by stabilization of β-catenin in *OC-Cre:Catnb* ^*lox/*+^ (*OC-Catnb*) mice^13^. Since Osx has been reported to be a key regulator in cellular cementum formation^17^, we hypothesized that the stabilized β-catenin might utilize Osx to drive excessive cementum formation in cementogenesis. To test this hypothesis, we generated and analyzed *OC-Cre:Catnb* ^*lox/*+^:*Osx* ^*fl/fl*^ (*OC-Catnb:Osx*) mice, which correspond to *OC-Catnb* mice with inactivation of *Osx*. Thus, stabilization of β-catenin is constitutively induced in the same *OC-*expressing cells. To analyze the role of each gene individually, we compared the double mutants with their wild type (WT) counterparts and with their corresponding single gene mutants, i.e., *OC-Catnb* and *OC-Cre:Osx* ^*fl/fl*^ (*OC-Osx*). The molars of *OC-Catnb* mice at postnatal week 6 (P6W) had a thicker cellular cementum layer on the overall root surface, including the cervical region with malformed dentin. Histological images of the cementum layer are shown in Fig. 1a (middle) and b. In addition, the molars of *OC-Catnb* mice exhibited much thicker apical cellular cementum compared to those of WT mice [Fig. 1a (bottom) and c]. In contrast, the molars of *OC-Osx* mice had thin interradicular dentin and a smaller apical cementum layer, with no remarkable change of acellular cementum at P6W [Fig. 1a (bottom) and c]. Strikingly, the molars of *OC-Catnb:Osx* mice exhibited dramatically restored morphology. Specifically, they had thinner acellular cementum, without the excessive irregular cellular cementum observed in *OC-Catnb* mice and without any root dentin malformation, at the same age. The mean cervical cementum thickness was slightly thicker in molars from *OC-Catnb:Osx* mice than in molars from *OC-Osx* mice (Fig. 1b). This cementum-forming pattern at the cervical region of roots was confirmed by observation of cervical cementum surfaces with scanning electron microscopy (SEM) (Supplementary Figure 1a). Detailed histological analysis of *OC-Catnb:Osx* molars revealed that the excessive cellular cementum located at the cervical region of the root had almost disappeared, no cementocyte-like cells were found within the matrix (Fig. 1a, middle), and the apical cellular cementum was much thinner, as in the *OC-Osx* molars (Fig. 1c). Root dentin was severely disturbed in the *OC-Catnb* molars, as evidenced by their uneven dentin border, which probably reflects an altered composition and mineralization. Interestingly, however, this malformation of root dentin was still observed in *OC-Catnb:Osx* molars, as it was observed at an earlier stage (P28) of tooth development in *OC-Catnb:Osx* mice (Supplementary Figure 1b). Although Osx clearly has a major role in dentin formation, these results highlight the special importance of Osx in the spatiotemporal regulation of cementum formation during tooth development. These results strongly suggest that Wnt/β-catenin signaling activated by stabilized β-catenin regulates cellular cementum formation via Osx.

### Stabilized β-catenin induces *Osx* expression in cementogenesis

**Figure 2:**
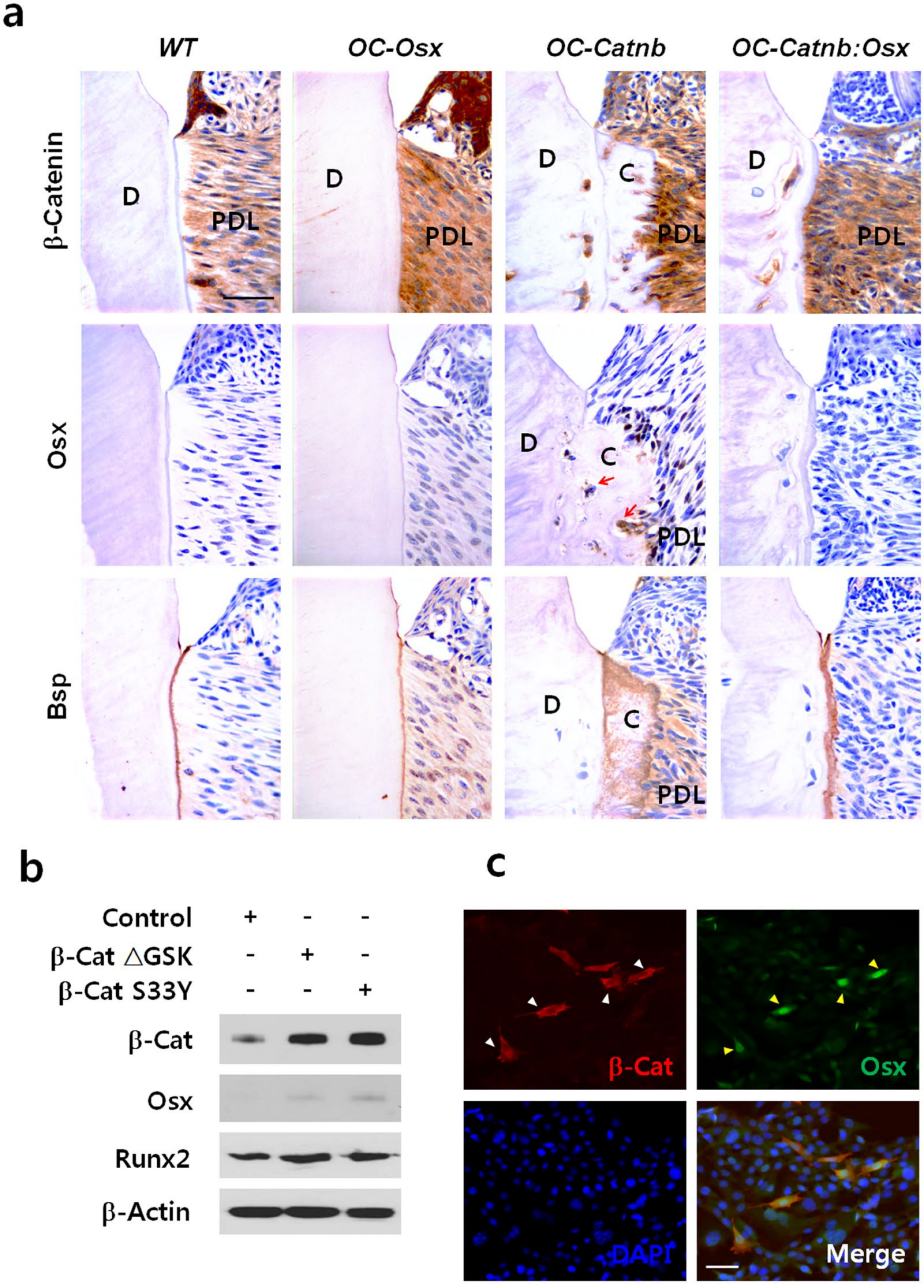
Stabilized β-catenin induces Osx expression in cementogenesis. (**a**) Immunohistochemical staining of the cervical cementum region of the distal root of the mandibular first molar in each group at P6W. Red arrows indicate strong Osx expression in cementoblasts and cementocyte-like cells in the excessive cementum in *OC-Catnb* mice. Scale bars, 20 μm. D, dentin; PDL, periodontal ligament; C, cementum. (**b**) The protein levels of β-catenin (β-Cat), Osx, and Runx2 were analyzed and compared by Western blotting. OCCM-30 cells were treated with OM for 1 day after transduction with retroviruses expressing two types of constitutively active mouse β-catenin, β-Cat S33Y, and β-Cat GSK. Samples shown are from the same experiment, and the gels/blots were processed under the same experimental conditions. β-Actin was used as a loading control. Cropped images are displayed here; the original full-size blots are presented in Supplementary Figure 2c. (**c**) Immunocytochemical staining was performed to look for colocalization of nuclear Osx and stabilized β-catenin. OCCM-30 cells were treated for 1 day with OM after transient transfection with plasmids driving the expression of β-Cat GSK. White arrowheads (β-catenin) and red arrowheads (Osx) indicate regions of colocalization of overexpressed stabilized β-catenin and nuclear Osx in the same cells. Nuclei were counterstained with DAPI. Scale bars, 50 μm.

As demonstrated with genetic mouse models with conditional disruption of *Osx* and overexpression of *Osx* in the dental mesenchyme^17^, Osx plays pivotal roles in cementogenesis. Moreover, the role of Osx is positively regulated by its expression level. In addition, Osx is probably a central molecule for controlling cementogenesis through numerous signaling pathways^18^. Thus, we examined Osx expression in molar tissue from P6W mice by immunohistochemical analysis. Consistent with our hypothesis, Osx was highly expressed in cells entrapped in the ectopically increased cellular cementum mass of *OC-Catnb* molars, which also showed stabilized β-catenin and broad Bsp staining in the cervical cementum (Fig. 2a). To determine whether stabilized β-catenin could induce the expression of Osx *in vitro*, we analyzed Tcf/Lef binding activities of two different active forms of mouse *β-catenin*, β-Cat S33Y and β-Cat GSK,^19, 20^ by using FOPflash/TOPflash luciferase reporters. As shown in Supplementary Figure 2a, the result of luciferase reporter assay indicates a definite elevation in Tcf/Lef binding activity of β-catenin by both of mutated mouse *β-catenin*. Based on this result, we transduced OCCM-30 cementoblast-like cells with retroviruses expressing these active forms of mouse *β-catenin*. As shown in Fig. 2b, both forms of stabilized β-catenin induced higher expression of Osx in OCCM-30 cells compared with control cells. However, expression of Runx2, a well-known Osx inducer in osteoblastogenesis^15^, was not as affected in transduced OCCM-30 cells expressing stabilized β-catenin. The stabilized β-catenin clearly induced Osx expression, as demonstrated by immunocytochemical staining. Specifically, nuclear Osx and the stabilized β-catenin colocalized after transfection of β-Cat GSK (Fig. 2c). In addition, the induction of Osx expression by β-Cat S33Y resulted in the upregulation of extracellular matrix genes including *OC*, *bone sialoprotein* (*Bsp*), and *dentin matrix protein 1* (*Dmp1*) in fully differentiated (i.e., after 4 days in osteogenic medium) OCCM-30 cells (Supplementary Figure 2b).

### Stabilized β-catenin transactivates the mouse *Osx* promoter in cementoblasts

**Figure 3:**
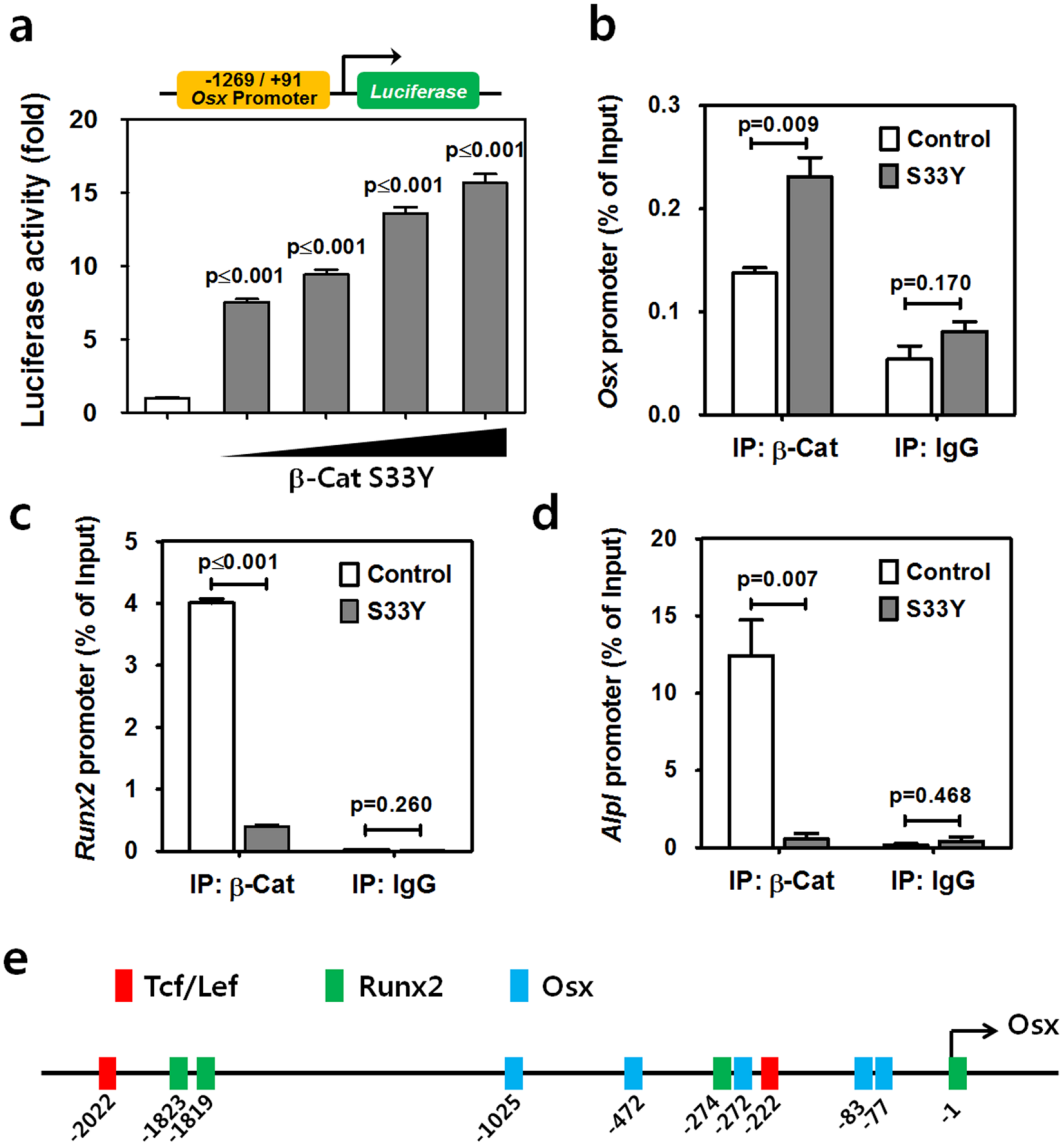
Stabilized β-catenin transactivates the mouse *Osx* promoter in cementoblasts. (**a**) Luciferase activity driven by the *Osx* (−1269/+91) promoter was analyzed using OCCM-30 cells treated with OM for 1 day after transduction with β-Cat S33Y. Gradually increasing concentrations of construct were used for transductions. The designated p-values indicate significant differences between the groups and the negative control. (**b**–**d**) ChIP-qPCR analysis of the *Osx*, *Runx2*, and *Alpl* promoters was performed using an anti-β-catenin antibody and chromatin from OCCM-30 cells treated with OM for 1 day after retroviral transduction with virus expressing β-Cat S33Y. Data represent mean ± SD of three measurements in each group. Significance was assigned for p-values as indicated. (**e**) The putative binding sites for Tcf/Lef (CTTTGGG) were exhibited with the consensus sites for *Runx2* (TCCCAC/AACCACA) and a preferred *Osx* binding site (CCACCC) in the −2036/ + 66 mouse *Osx* promoter.

β-Catenin is an established transcriptional activator^21, 22^. We thus investigated whether β-catenin transactivates the mouse *Osx* gene promoter. Interestingly, luciferase activity driven by the *Osx* promoter (−1269/+91) was increased up to 15-fold by transduction of β-Cat S33Y; this effect occurred in a concentration-dependent manner (Fig. 3a). The clear correlation between the amount of stabilized β-catenin and the promoter activity of *Osx* suggests that stabilized β-catenin can induce OSX expression by driving *Osx* expression. To examine whether stabilized β-catenin regulates transcription by binding directly to the promoter region of *Osx*, we performed chromatin immunoprecipitation (ChIP)-qPCR analysis using OCCM-30 cells. As shown in Fig. 3b, significantly increased recruitment of β-catenin to the regulatory regions of *Osx* was observed in OCCM-30 cells transduced with retrovirus expressing β-Cat S33Y compared with control cells. However, no association with the regulatory regions of *Runx2* (Fig. 3c) and *Alpl* (Fig. 3d) was detected in the same experimental conditions. Additionally, putative transcription factor binding sites in the mouse *Osx* promoter sequence (GenBank DQ229136) were screened. Putative binding sites for Tcf/Lefs (CTTTGGG) in the −2036/+66 mouse *Osx* promoter were exhibited with consensus sites for Runx2 (TCCCAC/AACCACA), a well-known upstream regulator of Osx, and a preferred Osx binding site (CCACCC) for self-driving expression^23–25^ in Fig. 3e. Moreover, two putative Tcf/Lef binding sites (CTTTGGG) were identified at −2022 and −222 among the four putative Runx2 binding sites and the five preferred Osx binding sites. These results suggest that β-catenin regulates its transcription, at least in part, by directly binding to the promoter region of *Osx* for cellular cementum formation.

### Stabilized β-catenin regulates cementogenesis in an Osx-dependent manner

**Figure 4:**
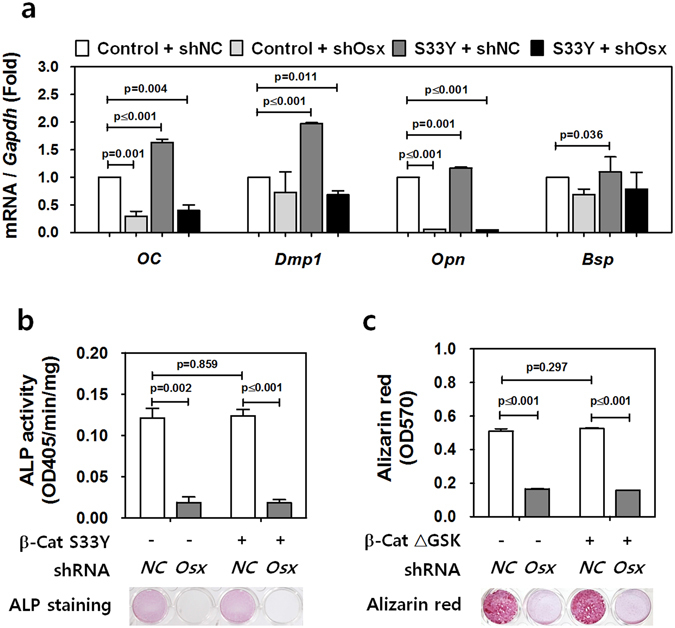
Stabilized β-catenin regulates cementogenesis in an Osx-dependent manner. (**a**) The transcript levels of various extracellular matrix genes including *Bsp*, *Opn*, *OC*, and *Dmp1* were analyzed by real-time qPCR. RNA was isolated from control (shNC) and *Osx*-ablated (shOsx) OCCM-30 cells treated with OM for 4 days after retroviral transduction of control and β-Cat S33Y-expressing viruses. Each designated p-value indicates a significant difference between the relevant group and the negative control (control retrovirus + shNC). (**b**) After treatment with OM for 4 days after transduction with control or β-Cat S33Y-expressing retrovirus, ALP activities was analyzed and displayed with ALP staining. (**c**) Mineralization in OCCM-30 cells expressing shNC or shOsx was analyzed by Alizarin red staining and quantified under the same experimental conditions as above. Data are presented as mean ± SD of three measurements of each group. Significance was assigned for p-values as indicated.

As shown in Fig. 1, cementoblasts with constitutive expression of stabilized β-catenin require Osx to produce excessive cellular cementum. Moreover, ablation of *Osx* prevents this abnormal accumulation and precludes the normal formation of apical cellular cementum. To further define the role of Osx in β-catenin-driven cementum formation, we compared various parameters of cementogenesis – differentiation, mineralization, and extracellular matrix secretion – in a similar *in vitro* genetic situation. As shown in Supplementary Figure 2b, the transcripts of all extracellular matrix genes tested were upregulated by β-Cat S33Y in OCCM-30 cells treated with OM for 4 days. However, unexpectedly, when *Osx* expression was silenced using small hairpin RNA (shRNA) specific for *Osx* (shOsx), real-time qPCR analysis of *OC*, *Bsp*, *Opn*, and *Dmp1* transcripts showed that the stabilized β-catenin could not upregulate these transcripts (Fig. 4a). This result suggests that stabilized β-catenin regulates matrix gene expression in an Osx-dependent manner in cementum formation. Alkaline phosphatase (ALP) activity and mineralization ability were not induced by constitutively active β-catenin significantly but exhibited a definite Osx-dependent regulation in OCCM-30 cells under the same conditions (Fig. 4b and c). Cumulatively, these observations demonstrate that stabilized β-catenin regulates cementogenesis in an Osx-dependent manner *in vitro*, as well as in genetic animal models.

### Osx regulates Lef1 expression in cementoblasts

**Figure 5:**
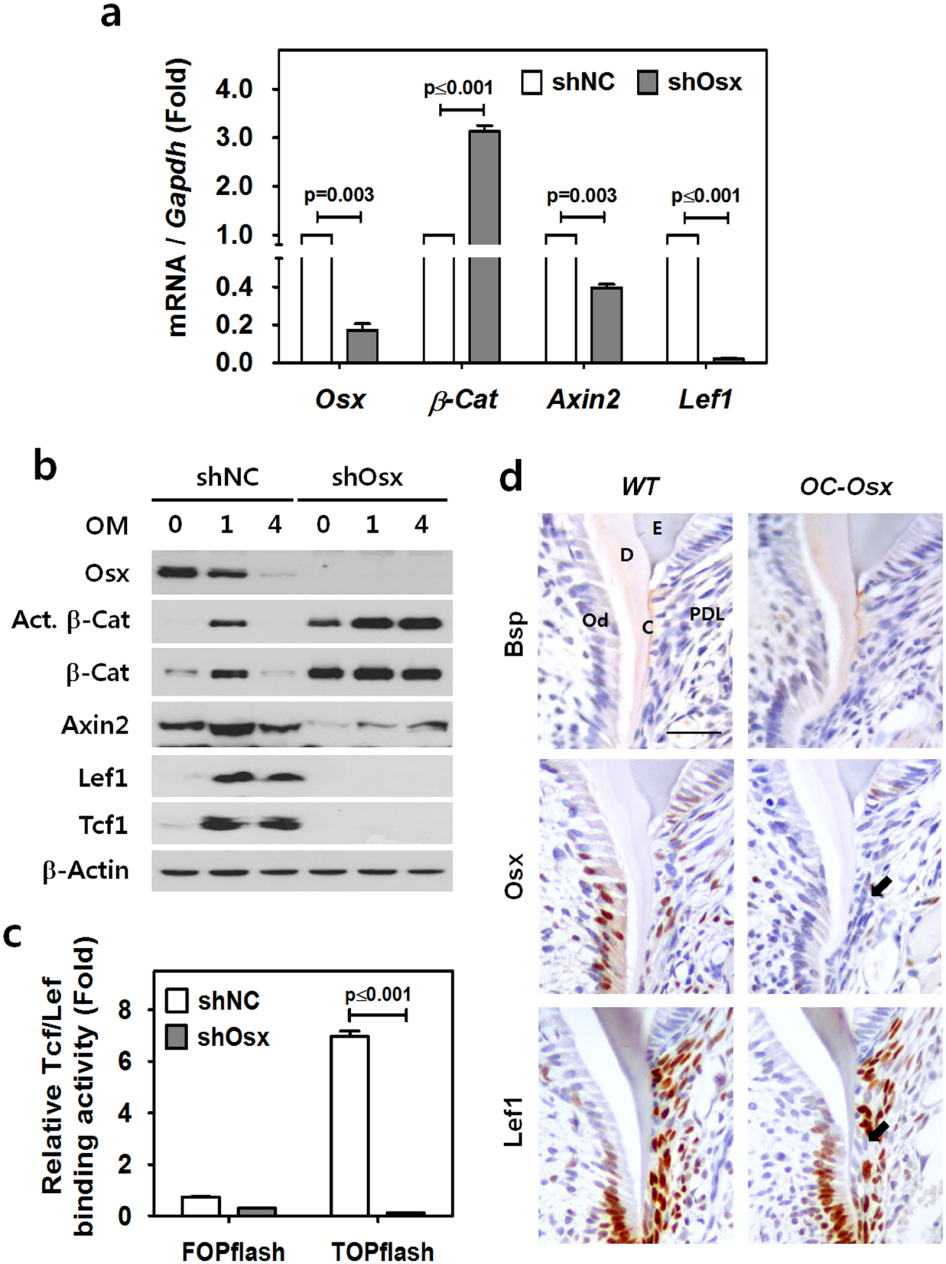
Osx regulates Lef1 expression in cementoblasts. (**a**) The transcript levels of genes associated with Wnt/β-catenin signaling, including *β-Cat*, *Axin2* and *Lef1*, were analyzed by real-time qPCR. RNA was isolated from control (shNC) and Osx-ablated (shOsx) OCCM-30 cells treated with OM for 1 day. Data are presented as mean ± SD of three measurements in each group. Significance was assigned for p-values as indicated. (**b**) The protein levels of Osx, the activated form of β-catenin lacking phosphorylation at S33/37/Thr41 (Act. β-Cat), β-catenin (β-Cat), Axin2, Lef1 and Tcf1 were analyzed and compared by Western blotting. Lysates were generated from OCCM-30 cells expressing shOsx or shNC at 0, 1, and 4 days after OM treatment. The samples shown were derived from the same experiment, and all gels/blots were processed under the same experimental conditions. β-Actin was used as a loading control. Cropped images are displayed here; the original full-size blots are presented in Supplementary Figure 3b. (**c**) Tcf/Lef binding activities were analyzed by FOPflash/TOPflash luciferase reporters in OCCM-30 cells expressing shNC or shOsx after treatment with OM for 1 day. Data are presented as mean ± SD of three measurements in each group. Significance was assigned for p-values as indicated. (**d**) Immunohistochemical staining of Bsp, Osx, and Lef1 at the cervical cementum region of the distal root of the mandibular first molars in WT and *OC-Osx* mice at P10. Black arrows indicate areas of reduced Lef1 expression and ablated Osx expression in cementoblasts from *OC-Osx* mice. Scale bars, 20 μm. E, enamel; D, dentin; Od, odontoblasts; PDL, periodontal ligament; C, cementum.

To address the role of Osx in the relationship between β-catenin and Osx in cementogenesis, we analyzed the expression of genes associated with Wnt/β-catenin signaling in OCCM-30 cells lacking *Osx* (shOsx) and in their corresponding control shRNA counterparts (shNC) by real-time qPCR (Fig. 5a). Interestingly, the transcript level of *Axin2*, a direct target of the Wnt/β-catenin/Tcf/Lef pathway^26^, was downregulated by ablation of *Osx*, even with the highly increased transcript level of *β-catenin* (*β-Cat*) in cementoblasts. Most interestingly, the transcript level of *Lef1* was remarkably decreased by ablation of *Osx* in cementoblasts. Furthermore, we also observed corresponding changes of these genes with ectopic overexpression of mouse *Osx*. Interestingly, the transcript levels of *Axin2* and *Lef1* were slightly increased by overexpression of *Osx*, whereas the level of *β-Cat* was not changed compared to the level in control cells (Supplementary Figure 3a). The molecular changes of the genes associated with Wnt/β-catenin signaling were also confirmed on the protein level by Western blotting. To this end, lysates of OCCM-30 cells transfected with shOsx or shNC and treated with OM for 1 day (short-term differentiation) or 4 days (full-term differentiation) were examined (Fig. 5b). Interestingly, the total amount of active β-catenin (non-phosphorylated S33/37/Thr41) in OCCM-30 cells lacking *Osx* (shOsx) gradually increased as differentiation proceeded. In contrast, control cells (shNC) only exhibited upregulation of active β-catenin with short-term differentiation. However, the protein level of Axin2 remained low and Lef1 and Tcf1 were undetectable in *Osx*-ablated OCCM-30 cells (shOsx). In contrast, control cells (shNC) exhibited higher overall levels of Axin2, Lef1 and Tcf1 detected during differentiation. Correspondingly, Tcf/Lef binding activity was almost undetectable in *Osx*-ablated OCCM-30 cells (shOsx) compared to control (shNC) cells, as demonstrated using FOPflash/TOPflash luciferase reporters (Fig. 5c). In addition, analysis of OCCM-30 cells transfected with β-Cat GSK showed that the loss of Tcf/Lef binding activity mediated by *Osx* ablation was partially recovered by stimulation with constitutively active β-catenin (Supplementary Figure 4). Thus, it is likely that Tcf/Lef binding activity is predominantly Osx-dependent in cementoblasts. To examine Lef1 expression in *Osx*-ablated cementum tissue, we performed immunohistochemical staining of molars from *OC-Osx* and WT mice at postnatal day 10 (P10). Positive BSP staining was observed in the cervical cementum line close to the cemento-enamel junction in both mouse lines. Osx and Lef1 were expressed in the cementoblasts, as well as in odontoblasts and some periodontal ligament (PDL) cells at the growing roots of WT molars. However, the cementoblasts in close proximity to the newly formed cementum lining of *OC-Osx* molars did not exhibit staining of Osx or Lef1 (Fig. 5d). Taken together, these results suggest that Osx regulates Tcf/Lef binding activity by controlling Lef1 expression in cementogenesis. In addition, to address the contradictory results of increased β-catenin protein level with lower transcriptional activity of β-catenin in *Osx*-ablated cells, we also investigated whether loss of *Osx* impairs nuclear translocation of β-catenin. As shown in Supplementary Figure 5a, however, there was no sign of impairment in nuclear translocation of β-catenin in *Osx*-ablated OCCM-30 cells (shOsx). These results strongly suggest that the accumulated β-catenin in *Osx*-ablated OCCM-30 cells was still non-phosphorylated and stable against ubiquitination but inactive for transcription.

### Osx regulates cementogenesis through Tcf/Lef

**Figure 6:**
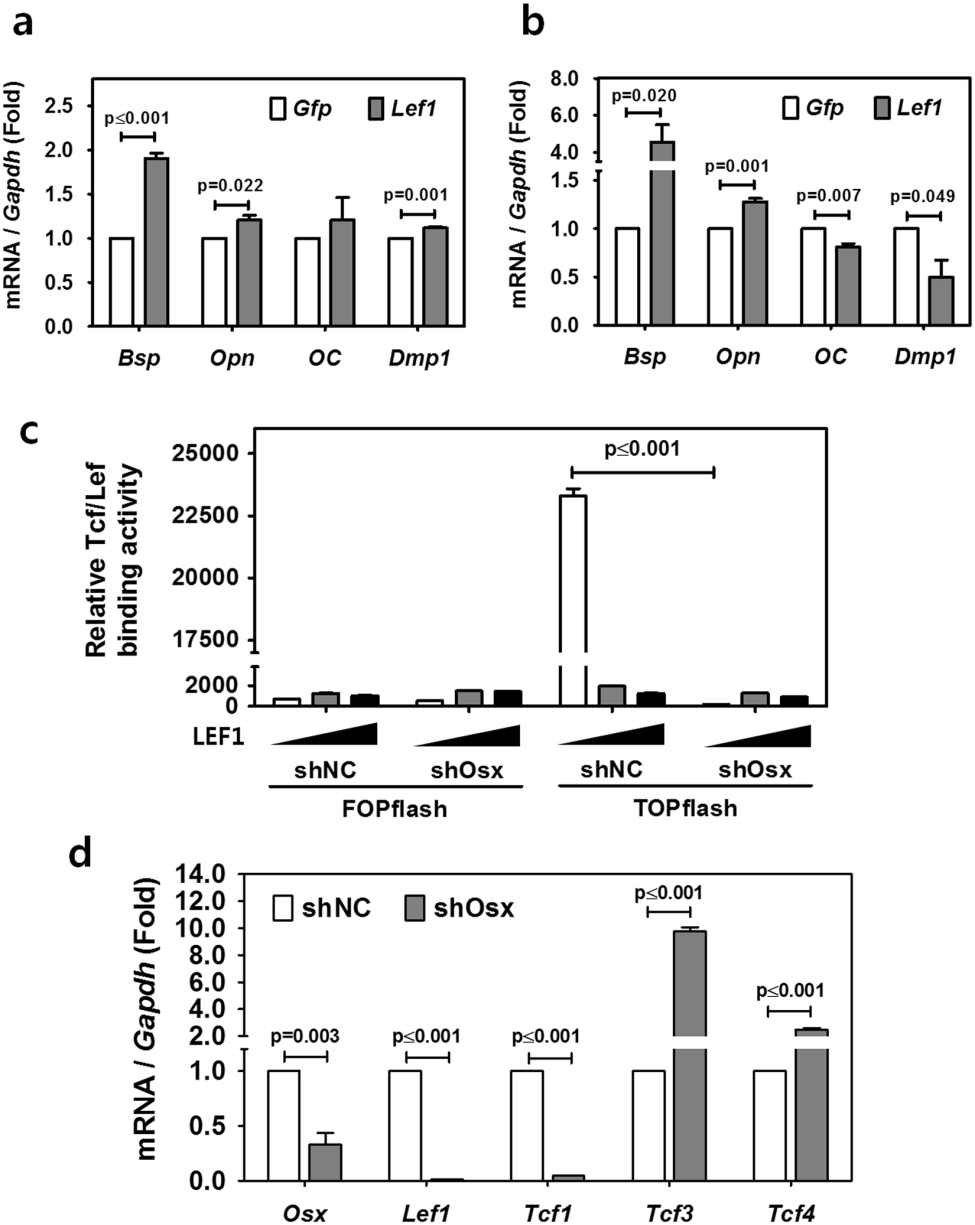
Osx regulates cementogenesis through Tcf/Lef. The transcript levels of various extracellular matrix genes including *Bsp*, *Opn*, *OC*, and *Dmp1* were analyzed by real-time qPCR. RNA was isolated from (**a**) OCCM-30 cells and (**b**) *Osx*-ablated (shOsx) OCCM-30 cells treated with OM for 4 days after transient transfection of plasmids driving the expression of *Gfp* (as a control) and mouse *Lef1*. (**c**) Tcf/Lef binding activities were analyzed by the FOPflash/TOPflash luciferase reporter assay. OCCM-30 cells expressing shOsx or shNC were transiently transfected with gradually increasing concentrations of a plasmid driving the expression of *Lef1* and then treated with OM for 1 day. (**d**) The transcript levels of various genes in the Tcf family, including *Lef1*, *Tcf1*, *Tcf3* and *Tcf4*, were analyzed by real-time qPCR. RNA was isolated from OCCM-30 cells expressing shOsx or shNC after treatment with OM for 1 day. All data are presented as mean ± SD of three measurements in each group. Significance was assigned for p-values as indicated.

Tcf/Lef transcription factors are the major endpoint mediators of Wnt/β-catenin signaling^9^. Because of the importance of Lef1 in Tcf/Lef binding activity^21^, we next examined whether Tcf/Lef binding activity could be recovered by Lef1 introduction into *Osx*-ablated OCCM-30 cells. LEF1 expression was achieved by transient transfection of mouse *Lef1* into OCCM-30 cells expressing control or shOsx. After confirming the expression of each transfected gene on the protein level by Western blotting (Supplementary Figure 6a), we evaluated the mRNA expression levels of cementoblastic extracellular matrix genes such as *Bsp*, *Opn*, *OC*, and *Dmp1* by real-time qPCR. As shown in Fig. 6a, at full-term differentiation, all analyzed transcripts of extracellular matrix genes were modestly upregulated by transfection of *Lef1* into OCCM-30 cells compared to the control gene (*Gfp*). However, as shown in Fig. 6b, not all tested transcripts were recovered by Lef1 overexpression in *Osx*-ablated OCCM-30 cells (shOsx). Compared to the control gene (*Gfp*), the transcriptional levels of *Bsp* and *Opn* were significantly increased by Lef1 overexpression in *Osx*-ablated OCCM-30 cells (shOsx), whereas those of *OC* and *Dmp1* were decreased by Lef1 overexpression. To examine whether Tcf/Lef binding activity could be recovered by Lef1 overexpression, we next analyzed FOPflash/TOPflash luciferase reporter activity in OCCM-30 cells expressing control or Osx-targeting (shOsx) shRNA. Unexpectedly, Tcf/Lef binding activities in control OCCM-30 cells (shNC) were greatly decreased by transfection of Lef1 (Fig. 6c). Also contrary to our expectations, Tcf/Lef binding activities in OCCM-30-shOsx cells were not significantly altered by Lef1 transfection, regardless of the amount of transfected DNA. To further address the role of Osx in Tcf/Lef activity, we extended our analysis to other Tcf family members. Real-time qPCR was performed to examine the mRNA expression of *Tcf1*, *Tcf3*, and *Tcf4* and to compare with them with that of *Lef1*. As shown in Fig. 6d, the transcripts of *Lef1* and *Tcf1*, two activators of Wnt target genes^9, 27^, were downregulated by ablation of *Osx* to as low as 0.01-fold and 0.05-fold, respectively, the levels in control (shNC) cells. Interestingly, however, the transcript level of *Tcf3*, a repressor of Wnt target genes^10, 28^, was increased by as much as 9.8-fold by ablation of *Osx*. In addition, the transcript level of *Tcf4* was also increased 2.5-fold by ablation of *Osx*. The full-length β-catenin-binding form of Tcf4 is sometimes associated with target gene repression^29^. When Osx was overexpressed via transfection of *Osx* into OCCM-30 cells, all transcripts of Tcf members were slightly upregulated compared to the control gene (*Gfp*) when assessed at full-term differentiation (Supplementary Figure 7). Taken together, these results strongly suggest that Osx regulates cementogenesis by controlling the expression of Lef1 along with all other Tcf/Lefs, and that the various Tcf/Lefs are involved in specific mechanisms.

## Discussion

To develop new tissue regeneration strategies, it is important to identify how developmental cues and regulatory factors are integrated to accommodate the requirements for biological control of cell differentiation and tissue formation. In this study, using both *in vivo* and *in vitro* approaches, we addressed the interaction of two key signals for cementogenesis: the Wnt/β-catenin pathway, which contributes to tooth morphogenesis^30–32^ and dental hard tissue formation including cementum^13, 32^, and the transcription factor Osx, which is essential for cementum formation^17, 18, 33^. Although Osx has an established central role in cementogenesis, the upstream and downstream signaling pathways that intersect with Osx and the interactions of Osx with other signaling molecules in cementogenesis are largely unknown. To understand the relationship between β-catenin signaling and Osx during cementum formation, we performed mechanistic studies to answer three main questions building on previous discoveries in mouse models: (1) What is the mechanism by which cementoblasts constitutively expressing stabilized β-catenin form excessive cellular cementum, such as that observed in *OC-Catnb* mice^13^? (2) At which step of cementogenesis do cementoblasts constitutively expressing stabilized β-catenin require Osx for cellular cementum formation, as observed in *OC-Catnb:Osx* mutant mice? (3) What is the mechanism by which Osx regulates β-catenin activity during cementogenesis?

Osx plays pivotal roles in cementogenesis and is a central molecule controlling cementogenesis through numerous signaling pathways. Histological analysis revealed that Osx expression was increased in the excessive cellular cementum in molars from *OC-Catnb* mice. In addition, stabilized β-catenin induced Osx expression in OCCM-30 cementoblast-like cells *in vitro*. Our ChIP analysis indicated that Osx expression is likely induced by direct regulation of the *Osx* promoter. However, indirect pathways are also likely to be involved in Osx induction. In agreement with our hypothesis, β-catenin signaling has been shown to upregulate Osx expression in osseous cells using human pre-osteoblastic and bone marrow stromal cells^34^. Regarding the mechanism, it was suggested that β-catenin signaling upregulates Osx expression by transactivating the *Osx* promoter, mainly through increased c-Jun binding at a putative c-Jun binding site. In addition to Osx induction, canonical Wnt signaling has been shown to promote osteogenesis. Specifically, the *Runx2* gene is a direct target of the canonical Wnt/β-catenin signaling pathway via Tcf1^23^. Cementoblasts share many properties with osteoblasts; therefore, similar regulatory mechanisms may be involved in control of tissue formation by Runx2 and Osx^1, 2^. However, based on the effects observed after blocking TGF-β^18^ and Wnt/β-catenin signaling, the regulation of Osx expression is likely more critical than that of Runx2 during cementogenesis. In support of this idea, the expression level of Osx was sensitively altered by the modulation of TGF-β or Wnt/β-catenin signaling, while the gross level of Runx2 was not altered.

Using *OC-Catnb:Osx* transgenic mice, we next addressed whether cementoblasts constitutively expressing stabilized β-catenin require Osx for cementogenesis. We found that cementoblasts constitutively expressing stabilized β-catenin require Osx to produce excessive cellular cementum, and that ablation of Osx prevents this abnormal accumulation and precludes the normal formation of apical cellular cementum. We performed protein expression profiling to determine the kinetics of this phenomenon in OCCM-30 cementoblast-like cells *in vitro* and found that Wnt/β-catenin signaling appears to regulate cell proliferation in the early stages. Later, i.e., after differentiation, Wnt/β-catenin signaling contributes to further matrix secretion during cementogenesis^18^. These *in vitro* experimental results clearly show that cementogenesis is regulated in an Osx-dependent manner in the presence of stabilized β-catenin, a finding consistent with the results using our transgenic mice in which Osx expression is controlled by a stage-specific Cre recombinase in OC-expressing cells. Taken together, these results strongly suggest that stabilized β-catenin induces cementoblast differentiation by triggering Osx expression and further regulates cementogenesis following matrix secretion and mineralization in an Osx-dependent manner. As exhibited *in vitro* data with *Osx*-silenced cementoblast-like cells (shOsx) in Fig. 4, various parameters for differentiation, mineralization, and extracellular matrix secretion indicate a clear Osx-dependent cementogenesis. However, not all the tested parameters of cementogenesis are positively dependent on β-Cat, probably due to its multitasking roles. Furthermore, our finding that the Tcf/Lef binding activity lost after *Osx* ablation was only partially recovered by stimulation with constitutively active β-catenin supports the idea that Tcf/Lef binding activity is also largely Osx-dependent in cementoblasts.

To gain insight into the mechanism by which Osx regulates β-catenin activity during cementogenesis, we analyzed the nuclear translocation of β-catenin and the expression of Tcf/Lef genes with *Osx* ablation. The results strongly imply that extraordinarily low levels of Tcf1 and Lef1 in *Osx*-ablated cells are one of the major mechanisms for impaired β-catenin activity even with its higher protein level. The nuclear mediators most closely associated with Wnt/β-catenin action are the Tcf/Lefs, a high-mobility group of DNA-binding proteins with multiple domains for protein interaction and regulation^9^. Lef1 is one of four transcription factors in the Tcf family^21^. Since we found that the protein and mRNA expression levels of Lef1 were very low in *Osx*-ablated OCCM-30 cells and that Tcf/Lef binding activity was also markedly reduced, we examined whether the Tcf/Lef binding activity lost after *Osx* ablation could be recovered by ectopic overexpression of Lef1 in *Osx*-ablated OCCM-30 cells. Some matrix transcripts such as *Bsp* were remarkablely recovered by ectopic overexpression of Lef1 in *Osx*-ablated OCCM-30 cells. However, unexpectedly, overexpression of Lef1 could not rescue all defects, including the lost TCF/LEF binding activity. These findings reflect that, in cementogenesis, complicated pathways downstream of Osx acting through Lef1–such as crosstalk between Lef1 and other negative factors, or other factors regulated by Osx–affect Tcf/Lef binding activity, in addition to Lef1. To investigate the mechanisms by which Osx controls the effects of β-catenin on Tcf/Lef binding activity in cementogenesis, we extended our study to all Tcf family members in *Osx*-ablated cementoblasts. One of our most interesting and novel findings is that Osx differentially regulates Tcf/Lef family members in cementum formation. This finding supports the hypothesis that Osx regulates cementogenesis through Wnt/β-catenin signaling by controlling Tcf/Lef expression andutilizes different Tcf/Lefs to act by distinct mechanisms. This explanation would account for the ability of Wnt/β-catenin signaling to elicit distinct responses at different time points and in different tissues. This type of response pattern depends critically upon the major regulator of the target tissue, such as Osx in the case of cementum, to control target gene expression in a highly context-dependent manner^35, 36^. For example, *Osx*-ablated cementoblasts have decreased levels of Lef1 and Tcf1 (activators of Wnt/β-catenin), but increased levels of some family member such as Tcf3, which appears to function primarily as a repressor of Wnt/β-catenin. This finding implies that Osx helps fine-tune the signal specificity and strength of Wnt/β-catenin signaling for cementogenesis through different Tcf/Lefs. However, the mechanism by which Osx regulates Tcf/Lefs in cementoblasts and the relevant roles of these proteins in downstream pathways of Osx in cementogenesis still need to be determined.

In summary, using *in vivo* and *in vitro* approaches, our study provides compelling evidence to support a mechanism in which Wnt/β-catenin promotes cementum formation through Tcf/Lef-mediated activation of the master cementogenic transcription factor Osx. Osx, in turn, regulates β-catenin activity by controlling Tcf/Lef expression during cementum formation. This is the first demonstration of a reciprocal interaction between β-catenin and Osx and the first report to show that a pathway downstream of Osx that signals through Tcf/Lefs is critical for cementogenesis during tooth development. Our report will potentially stimulate interest in local modulation of β-catenin and Osx and could ultimately be applied to develop therapies aimed at improving periodontal structure, including cementum regeneration.

## Materials and Methods

### Mouse strains

All procedures were performed in accordance with the National Institutes of Health Guidelines on the Use of Laboratory Animals. All experimental protocols and animal care methods were approved by the Animal Welfare Committee of Chonbuk National University. *Catnb* ^*lox(ex3)/lox(ex3)*^ (*Catnb* ^*lox/lox*^), *Osx*-floxed allele (*Osx* ^*fl/fl*^), and *OC-Cre* mice have been previously described^14, 37, 38^. *OC-Cre:Catnb* ^*lox/*^+ (*OC-Catnb*) and *OC-Cre:Osx* ^*fl/fl*^ (*OC-Osx*) mice were generated as described^13, 16^. To generate *OC-Cre:Catnb* ^*lox/*^+:*Osx* ^*fl/fl*^ (*OC-Catnb:Osx*) mice, *OC-Cre;Osx* ^*fl/*^+ (control) mice were crossed with *Catnb* ^*lox/lox*^:*Osx* ^*fl/fl*^ mice, and the offspring were genotyped by polymerase chain reaction (PCR) analysis using previously described primers^14, 37, 38^.

### Immunohistochemistry and histomorphometry

For immunostaining, sections were treated with 3% hydrogen peroxide and incubated with rabbit polyclonal antibodies against β-catenin (1:200; Thermo Scientific, Fremont, CA, USA), Osx (1:200; Abcam, Cambridge, MA, USA), and bone sialoprotein (Bsp, 1:50; Abcam). The Histostain Plus Rabbit Primary (DAB) kit (Zymed Laboratories, San Francisco, CA, USA) and goat ImmunoCruz staining system (Santa Cruz Biotechnology, Dallas, TX, USA) were used following the manufacturers’ instructions. Cervical cementum thickness was measured as the shortest vertical distance at a site 100 μm apical from the cemento–enamel junction in the mid-sagittal section of the mandibular first molar using the analySIS Pro imaging system (Soft Imaging System, Münster, Germany). The five starting points of the proximal line for measurement were randomly selected within the apical part of each root. Experiments were performed three times with representative slides from each group, and statistical analysis was performed to evaluate the significance of differences between values (p < 0.01). The average apical cementum area was calculated using the analySIS Pro imaging system. For this calculation, three measurements from five representative slides in each group were used; the analyzed tissue was harvested from 6-week-old mice.

### Cell culture

OCCM-30, a mouse cementoblast cell line, was kindly provided by Dr. Martha J. Somerman (National Institutes of Health, Bethesda, MD, USA) and cultured as described previously^39^. To induce cell differentiation and mineralization, 95% confluent cells were cultured in osteogenic medium (OM), which consisted of medium supplemented with 5% fetal bovine serum, 50 μg/ml ascorbic acid (Sigma Aldrich, St. Louis, MO, USA), and 10 mM β-glycerophosphate (Sigma Aldrich), for up to 4 days.

### Transfection and retroviral transduction

The luciferase *Osx* promoter plasmid −1269/+91 was kindly provided by Dr. Mark Nanes (Emory University, Atlanta, GA, USA). The TOPflash β-catenin reporter construct containing the Tcf/Lef binding sites and the FOPflash control reporter containing mutated Tcf/Lef binding sites were gifts from Randall Moon (Addgene plasmids #12456 and #12457). Plasmids driving the expression of mouse β-catenin deltaGSK (β-Cat GSK) and S33Y were gifts from Tannishtha Reya and Shinya Yamanaka, respectively (Addgene plasmids #14717 and #13371). Four serine and threonine residues (Ser-33, Ser-37, Thr-41, and Ser-45) of β-catenin in recognition sites for GSK-3β were changed to alanine in β-Cat GSK^40^. Flag-tagged mouse *Osx* (accession no. NM_130458) and Myc-tagged *Lef1* (accession no. BC057543) constructs in the pCMV6 backbone were purchased from OriGene Technologies (Rockville, MD, USA). A *Gfp* construct was also transfected as a control to analyze GFP protein production^18^. Transfection experiments were performed with Lipofectamine^TM^ LTX and PLUS Reagent (Invitrogen, New York, NY, USA) according to the manufacturer’s instructions. After 24 h, transfected cells were harvested for whole cell lysate preparation or cultured with OM for further differentiation. Retroviral particles harboring short hairpin RNA (shRNA) against mouse *Osx* (TG514041) or control shRNA (TR30013) (OriGene Technologies) were generated for stable cell lines with shRNA as described previously^18^. To establish stable cell lines by viral transductions, subconfluent OCCM-30 cells were transduced for 24 h with the viral particles expressing shRNA directed against mouse *Osx* or control shRNA in the presence of polybrene (4 μg/ml) (Sigma Aldrich). Forty-eight hours later, transduced OCCM-30 cells were selected using growth medium containing 10 µg/ml puromycin (Santa Cruz Biotechnology). For retroviral transduction of β-Cat GSK and S33Y into OCCM-30 cells, viral particles were generated by transfecting a 293T-based amphotropic retroviral packaging cell line, Phoenix, with the plasmids for 24 h using LipofectamineTM LTX and PLUS reagent (Invitrogen) according to the manufacturer’s instructions. Supernatants containing viral particles were collected between 48 after transfection, filtered through a 0.45-μm filter, and then used after 100-fold concentration using a Retro-X Concentrator (Clontech, Mountain View, CA, USA) according to the manufacturer’s instructions. Finally, cells were transduced by adding 25 μl of concentrated viral supernatant into one well of a 12-well plate where cell density is at 80–90% for 24 h in the presence of polybrene. This amount of viral particles is estimated to infect target cells at a MOI (multiplicity of infection) of 50.

### Chromatin immunoprecipitation (ChIP)

We performed ChIP as previously described^18^ (details are described in Supplementary Methods). The ChIP experiments used an anti-β-catenin rabbit polyclonal antibody (1:100; Thermo Scientific) and normal rabbit IgG (Santa Cruz Biotechnology). PCR primers are listed in Supplementary Table 1. Putative transcription factor binding sites in the mouse *Osx* gene promoter sequence were identified using online PROMO software (http://alggen.lsi.upc.es/cgi-bin/promo_v3/promo/), as previously described^41, 42^.

### Statistical analysis

Data are presented as mean ± standard deviation (SD) of three or more separate experiments, as indicated. Normal data with equal variance were analyzed using Student’s t-test or one-way analysis of variance with Tukey’s procedure. Significance was assigned for p-values as indicated.

## Electronic supplementary material

Supplementary Information
