# Supplementary material for: A Reciprocal Interaction between β-Catenin and Osterix in Cementogenesis
Source: Sci Rep. 2017 Aug 15;7:8160. doi: 10.1038/s41598-017-08607-5 (PMC5558006; doi:10.1038/s41598-017-08607-5)

Supplementary Information

A Reciprocal Interaction between β-Catenin and Osterix in Cementogenesis

Hwajung Choi[1], Tak-Heun Kim[1], Siqin Yang[1], Jeong-Chae Lee[1], Hyung-Keun You[2], Eui-Sic Cho[1],*

[1]Cluster for Craniofacial Development and Regeneration Research, Institute of Oral Biosciences, Chonbuk National University School of Dentistry, Jeonju 54896, South Korea

[2]Department of Periodontology, School of Dentistry, Wonkwang University, Iksan 54538, South Korea

[*] Author for correspondence

Eui-Sic Cho, DDS, PhD

Laboratory for Craniofacial Biology,

Chonbuk National University School of Dentistry

567 Baekje-Daero, Deokjin-Gu, Jeonju 54896, South Korea

Phone: 82- 63- 270- 4045, Fax: 82- 63- 270- 4004

E-mail: oasis@jbnu.ac.kr

Supplementary Table 1. Primer sequences for ChIP-qPCR

Gene	Sense	Antisense
Alpl	GGCTGGGACAGACAGAATGT	CTGCAACAGGCAGGGTAAC
Osx	CTCATTGGATCCGGAGTCTTCT	TGTCTGTAGGGATCCACCCTCTA
Runx2	TGGTAGGCAGTCCCACTTTAC	GGCTGGTAGTGACCTGCAGAG

Supplementary Table 2. Primer sequences for real-time qPCR

Gene	Sense	Antisense
Osx	TCTCCATCTGCCTGACTCCT	AGCGTATGGCTTCTTTGTGC
Bsp	AAAGTGAAGGAAAGCGACGA	GTTCCTTCTGCACCTGCTTC
Oc	ACCCTGGCTGCGCTCTGTCTCT	GATGCGTTTGTAGGCGGTCTTCA
Dmp1	AGTGAGTCATCAGAAGAAAGTCAAGC	CTATACTGGCCTCTGTCGTAGCC
Axin2	AAGAAGGAGACCGGTCACAG	GGTCCTGGGTAAATGGGTGA
Lef1	TTCAGGTACAGGTCCCAGAATG	AGTCGGCGCTTGCAGTAGA
β-Cat	GCCATCTGTGCTCTTCGTC	ACACCCTTCTACTATCTCCTCC
Tcf1	TACTATGAACTGGCCCGCAA	AGCACTGTCATCGGAAGGAA
Tcf3	TGAAGGAGATGAGGGCCAAG	TCTTCCCATAGTTGTCCCGG
Tcf4	CTTCACATGCAGCTGTACCC	GGCATTTCTTAGGAGCGCTC
Gapdh	TGCCCAGAACATCATCCCT	GGTCCTCAGTGTAGCCCAAG

Supplementary Methods

Tissue preparation and histology

For histologic analysis, mice were sacrificed and their mandibles were carefully dissected. The dissected tissues were fixed in 4% paraformaldehyde (PFA) and decalcified in 10% EDTA for 2 to 4 weeks at 4℃. The decalcified tissues were dehydrated through a graded ethanol series, embedded in paraffin, and sectioned at 5-μm thickness. Slides were stained with hematoxylin and eosin (H-E).

Scanning electron microscopy (SEM)

Mandibular first molars were isolated from mice at postnatal day 28 (P28) and fixed in 70% ethanol at room temperature for 24 h. The specimen surfaces were sputter-coated with platinum after drying and examined with a scanning electron microscope (JSM-6400; JEOL, Tokyo, Japan) using an acceleration voltage of 20 kV.

RNA preparation and real-time qPCR

Prior to RNA isolation, 5.0 x 10^5 cells were seeded in a 60 mm culture dish and cultured for 3 weeks under mineralization differentiation induction conditions. Total RNA was prepared using an RNeasy Mini kit (QIAGEN, Valencia, CA, USA) according to the manufacturer's instructions, and cDNA was synthesized from 2 μg of total RNA using Superscript II reverse transcriptase (Invitrogen). Real-time PCR was performed with SYBR Green PCR Master Mix (Applied Biosystems, Warrington, Cheshire, UK) following the manufacturer's protocols. Reaction conditions comprised 40 cycles of 15 sec of denaturation at 95℃ and 1

min of amplification at 60℃. All reactions were run in triplicate; expression was normalized to that of the housekeeping gene *glyceraldehyde-3-phosphate dehydrogenase* (*Gapdh*). Relative levels of transcript expression were quantified using the $\Delta\Delta Ct$ method, as previously described[18]. Relative mRNA expression was compared in a histogram. Specific primer sets used in the analysis are listed in Supplementary Table 2.

Luciferase activity

Luciferase activity was determined using the Dual-Luciferase reporter assay system (Promega, Madison, WI, USA) according to the manufacturer's instructions. Light intensity was measured with a luminometer. All measured luciferase activities were divided by the activity of the control reporter to normalize for transfection efficiency.

Western blot analysis

Proteins (30 μg) were dissolved in sample buffer and resolved by electrophoresis with a current of 25 mA for 2 h. Proteins were transferred from the SDS-PAGE gels onto PVDF membranes (Schleicher & Schuell, Dassel, Germany). Nonspecific binding sites on membranes were blocked for 1 h with 5% nonfat dry milk in PBS containing 0.05% Tween-20 (PBS-T) and incubated overnight with IgG antibodies against Osx (Santa Cruz Biotechnology), β-catenin (Thermo Scientific), Runx2 (Abcam), Axin2 (Abcam), active β-catenin (Cell Signaling), Lef1 (Cell Signaling), Tcf1 (Cell Signaling), GFP (Abcam), Myc (Cell Signaling), α-tubulin (Santa Cruz Biotechnology) and β-actin (Santa Cruz Biotechnology) diluted 5% nonfat dry milk in PBS-T buffer at 4 ℃. After washing, the membranes were incubated with horseradish peroxidase-conjugated anti-rabbit or anti-mouse

IgG antibodies (Santa Cruz Biotechnology) for 1 h. Immunoreactive bands were detected using an enhanced chemiluminescence system (Amersham Biosciences, Buckinghamshire, UK). Protein expression levels were analyzed with the ImageQuanT TL 1D gel analysis program (Amersham Biosciences). To extract cytoplasmic and nuclear proteins, cells were resuspended in buffer A [10 mM HEPES (pH 7.9), 1.5 mM $MgCl_2$, 10 mM KCl, 0.5 mM DTT, 0.5 mM PMSF, 0.4% Nonidet P-40 (v/v), and 1x protease inhibitor cocktail] for 20 min on ice. After centrifugation at 2,300 x g for 5 min, the supernatants were used as cytoplasmic extracts. The resulting pellets were resolved in buffer C [20 mM HEPES (pH 7.9), 420 mM NaCl, 1.5 mM $MgCl_2$, 0.2 mM EDTA, 0.5 mM DTT, 0.5 mM PMSF, and 1x protease inhibitor cocktail] for 30 min on ice. After centrifugation at 16,000 x g for 15 min, the supernatants were used as nuclear extracts.

Immunocytochemical staining

Cells were fixed in 4% paraformaldehyde in PBS at room temperature for 10 min. They were then permeabilized by incubation in 0.2% Triton X-100 for 10 min at room temperature, after which endogenous peroxidase activity was quenched by incubation in 3% H_2O_2 in the dark. After rinsing with PBS, nonspecific binding sites on the cells were blocked with 5% BSA in PBS for 30 min at room temperature. Cells were then incubated with primary antibodies against β-catenin (Thermo Scientific) and Osx (Santa Cruz Biotechnology) for 16 h at 4°C. Normal rabbit immunoglobulin (IgG, Santa Cruz Biotechnology) was used as a control primary antibody. Alexa Fluor®-conjugated secondary antibodies (Invitrogen) were used for detection. DAPI was used for counterstaining. Cell staining was evaluated using a fluorescence microscope (Carl Zeiss, Ostalbkreis, Germany).

Mineralization induction and alizarin red staining

To induce cell differentiation and mineralization, 95% confluent cells were cultured in osteogenic media (OM) consisting of DMEM supplemented with 5% FBS, 50 μg/ml ascorbic acid, and 10 mM β-glycerophosphate (Sigma Aldrich) for up to 4 days. Mineral nodule formation was observed by staining the cells with 40 mM alizarin red S (pH 4.2) after fixation with 4% PFA for 10 min. The amount of alizarin red S that bound to the minerals was quantified by destaining the samples in 10 mM sodium phosphate containing 10% cetylpyridinium chloride (pH 7.0) for 15 min at room temperature. The amount of alizarin red S in the destaining solution was measured at OD 562 nm.

Alkaline phosphatase (ALP) activity and staining

Alkaline phosphatase activity was quantitated using an assay based on the hydrolysis of p-nitrophenylphosphate (p-NPP) to p-nitrophenol (p-NP). Briefly, cell layers were washed twice with ice-cold phosphate-buffered saline (PBS) and lysed in 50 mM Tris–HCl buffer (pH 7.0) containing 1% (v/v) Triton X-100 (Sigma Aldrich) and 1 mM PMSF (Sigma Aldrich). Whole cell lysates were assayed by adding 1 mg/ml of pNPP substrate in 0.1 M glycine buffer (pH 10.4) containing 1 mM $ZnCl_2$ (Sigma Aldrich) and 1 mM $MgCl_2$ (Sigma Aldrich) to each tube for 15 min at 37 °C. Reactions were stopped by adding NaOH (final concentration 0.6 N), and the absorbance of each lysate was measured spectrophotometrically at 405 nm. Enzyme activity is expressed as OD405/min/mg of protein or as a percentage of the control lysate. The concentration of protein in each cell lysate was measured using a DC Protein Assay® (Bio-Rad Laboratories, Hercules, CA,

USA). For ALP staining, cells were fixed in 10% formalin, incubated with 0.1% Triton X-100 for 5 min, and then stained with a Leukocyte Alkaline Phosphatase kit (Sigma Aldrich) according to the manufacturer's protocol.

Chromatin immunoprecipitation (ChIP)

ChIP assays were performed using a ChIP assay kit from Millipore (Billerica, MA, USA) according to the manufacturer's protocol with minor modifications. In brief, cells were cross-linked with 1% formaldehyde for 5 min at room temperature. After a single wash in phosphate buffered saline (PBS), an aliquot of the cross-linked chromatin was sonicated and incubated overnight with anti-β-catenin antibodies (1:100; Thermo Scientific) or normal rabbit IgG. After incubation with protein G-conjugated agarose beads for 2 h, the beads were washed several times, and the DNA–protein complexes were eluted. The cross-links were reversed by overnight incubation at 65°C in the presence of 25 mM NaCl and subsequent digestion with RNase A and proteinase K, followed by purification of the ChIP-DNA. The ChIP-DNA was then amplified by qPCR; all reactions were carried out in triplicate. The total amounts of bound anti-β-catenin antibody were determined by expressing the amount of DNA obtained from each immunoprecipitation sample as a percentage of the total DNA.

Legends for Supplementary Figures

Supplementary Figure 1. *In vivo* regulation of cementum formation by stabilized β-catenin and Osx. (a) SEM analysis of the surface structure of the cervical cementum of mandibular first molars from WT, *OC-Osx*, *OC-Catnb*, and *OC-Catnb:Osx* mice analyzed at postnatal day 28 (P28). Scale bar, 5 μm. **(b)** H-E-stained sections of mandibular first molars from WT, *OC-Osx*, *OC-Catnb,* and *OC-Catnb:Osx* mice at P28. The malformation of root dentin observed in *OC-Catnb* molars was still observed in *OC-Catnb:Osx* molars. However, the thin acellular cervical cementum was dramatically restored in *OC-Catnb:Osx* molars, in contrast to the excessive cervical cellular cementum observed in *OC-Catnb* molars. Scale bars, 20 μm. D, dentin; AC, acellular cementum; CC, cellular cementum.

Supplementary Figure 2. Tcf/Lef binding activity of mutated mouse *β-catenin* and mRNA expression levels of genes associated with the extracellular matrix in OCCM-30 cells expressing stabilized β-catenin. (a) The Tcf/Lef binding activities of both types of mutated mouse *β-catenin* (β-Cat △GSK and β-Cat S33Y) were analyzed with FOPflash/TOPflash luciferase reporters. **(b)** The mRNA expressions of extracellular matrix genes were examined by real-time qPCR. RNA was isolated from OCCM-30 cells transduced with a retrovirus expressing control and β-Cat S33Y and then treated with OM for 4 days. Data are presented as mean ± SD of three measurements in each group. Significance was assigned for p-values as indicated. **(c)** Original full-size blots from Figure 2B. The following antibodies were used: anti-β-catenin (1:2000, RB-9035, Thermo Scientific), anti-Osx (1:500,

sc-22536, Santa Cruz Biotechnology), anti-Runx2 (1:2000, ab23981, Abcam), and anti-β-actin (1:2000, sc-1616R, Santa Cruz Biotechnology).

Supplementary Figure 3. mRNA expression levels of genes associated with Wnt/β-catenin signaling in OCCM-30 cells overexpressing *Osx*. (a) The mRNA expression levels of genes associated with Wnt/β-catenin signaling, including *β-Cat*, *Axin2*, and *Lef1*, were analyzed by real-time qPCR. RNA was isolated from OCCM-30 cells transiently transfected with *Osx* and then treated with OM for 1 day. A plasmid driving the expression of *Gfp* was transfected as a control. Data are presented as mean ± SD of three measurements in each group. Significance was assigned for p-values as indicated. **(b)** Original full-size blots from Figure 5b. The following antibodies were used: anti-Osx (1:500, sc-22536, Santa Cruz Biotechnology), anti-active β-catenin (1:1000, 8814, Cell Signaling), anti-β-catenin (1:2000, RB-9035, Thermo Scientific), anti-Axin2 (1:500, ab32197, Abcam), anti-Lef1 (1:1000, 2230, Cell Signaling), anti-Tcf1 (1:1000, 2203, Cell Signaling), and anti-β-actin (1:2000, sc-1616R, Santa Cruz Biotechnology).

Supplementary Figure 4. Regulation of Tcf/Lef binding activity by β-catenin and Osx. Tcf/Lef binding activities were analyzed by FOPflash/TOPflash luciferase reporters. The assay was performed using OCCM-30 cells expressing shOsx and shNC. Cells were transfected with plasmids driving the expression of *Gfp* (GFP) and *β-Cat △GSK* (△GSK) and then treated with OM for 1 day. Data are presented as mean ± SD of three measurements in each group. Each designated p-value indicates a significant difference between the given group and the negative control (shNC + GFP).

Supplementary Figure 5. Nuclear translocation of β-catenin in *Osx*-ablated OCCM-30 cells. (a) The protein levels of Osx, the activated form of β-catenin lacking phosphorylation at S33/37/Thr41 (Act. β-Cat), β-catenin (β-Cat), and α-Tubulin were analyzed with cytoplasmic (Cyto) and nuclear (Nucl) proteins and compared by Western blotting. Protein extracts were fractionated from OCCM-30 cells expressing shOsx or shNC at undifferentiated state. The samples shown were derived from the same experiment, and all gels/blots were processed under the same experimental conditions. α-Tubulin was used as a loading control. Cropped images are displayed here; the original full-size blots are presented below. **(b)** Original full-size blots from the experiment shown in (a). The following antibodies were used: anti-Osx (1:500, sc-22536, Santa Cruz Biotechnology), anti-active β-catenin (1:1000, 8814, Cell Signaling), anti-β-catenin (1:2000, RB-9035, Thermo Scientific), and anti-α-Tubulin (1:1000, sc-8035, Santa Cruz Biotechnology).

Supplementary Figure 6. Overexpression of Lef1 by transient transfection in *Osx*-ablated OCCM-30 cells. (a) The protein levels of Osx, GFP, Myc-tag (Myc), and Lef1 in lysates from OCCM-30 cells expressing shOsx or shNC and transiently transfected with a plasmid driving the expression of mouse *Lef1* were compared. A plasmid driving the expression of *Gfp* was transfected as a control. The samples shown were derived from the same experiment, and all gels/blots were processed under the same experimental conditions. β-actin was used as a loading control. Cropped images are displayed here; the original full-size blots are presented below. **(b)** Original full-size blots from the experiment shown in (a). The following antibodies were used: anti-Osx (1:500, sc-22536, Santa Cruz Biotechnology),

anti-GFP (1:2000, ab290, Abcam), anti-Myc (1:1000, 2276, Cell Signaling), anti-Lef1 (1:500, 2230, Cell Signaling), and anti-β-actin (1:2000, sc-1616R, Santa Cruz Biotechnology).

Supplementary Figure 7. mRNA expression levels of Tcf family genes in OCCM-30 cells overexpressing *Osx*. The mRNA expression levels of Tcf family genes including *Lef1*, *Tcf1*, *Tcf3*, and *Tcf4* were analyzed by real-time qPCR. RNA was isolated from OCCM-30 cells transiently transfected with a plasmid driving the expression of *Osx*. A plasmid driving the expression of *Gfp* was transfected as a control. Data are presented as mean ± SD of three measurements in each group. Significance was assigned for p-values as indicated.

Supplementary Figure 1

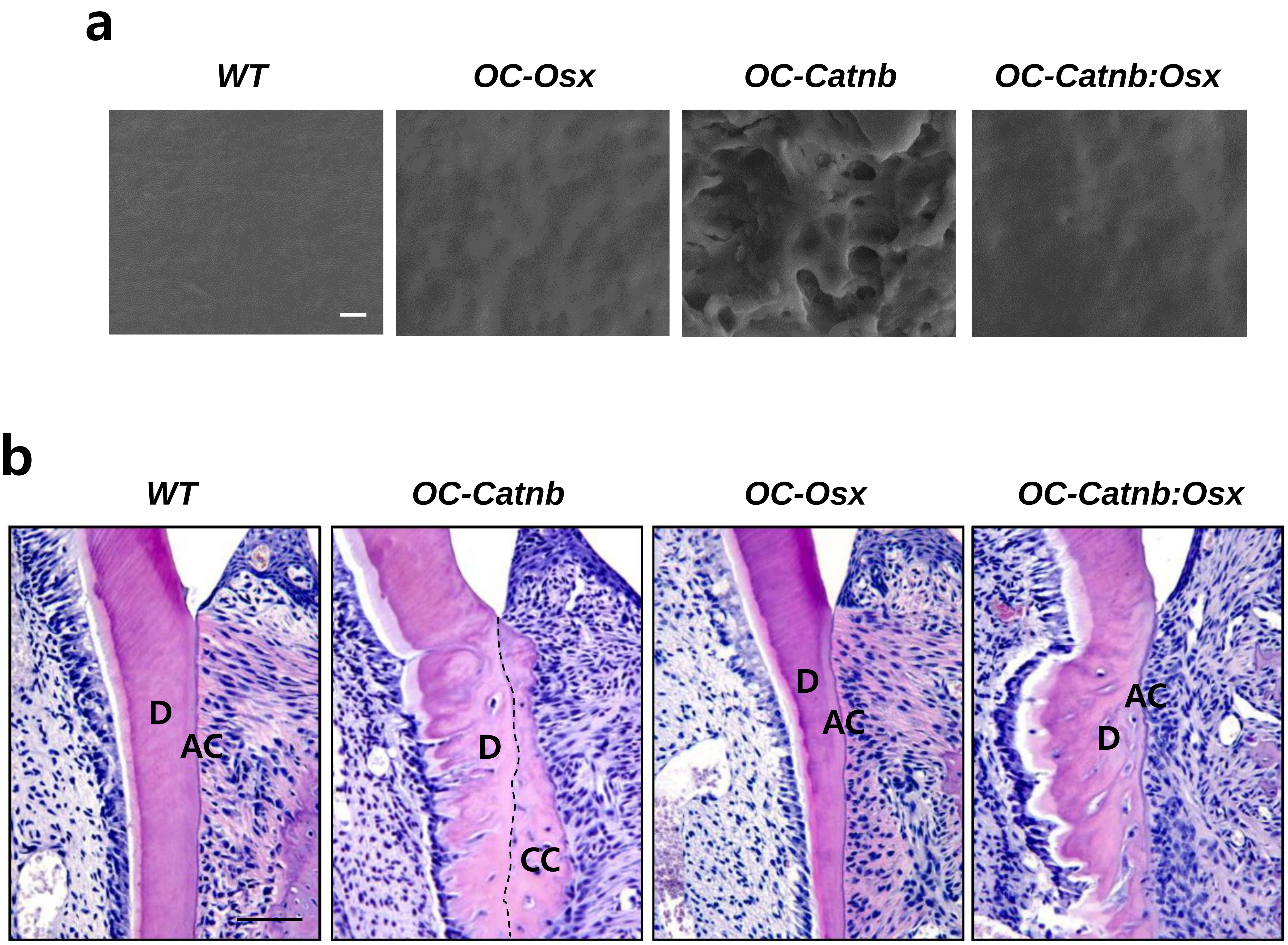

Supplementary Figure 2

a

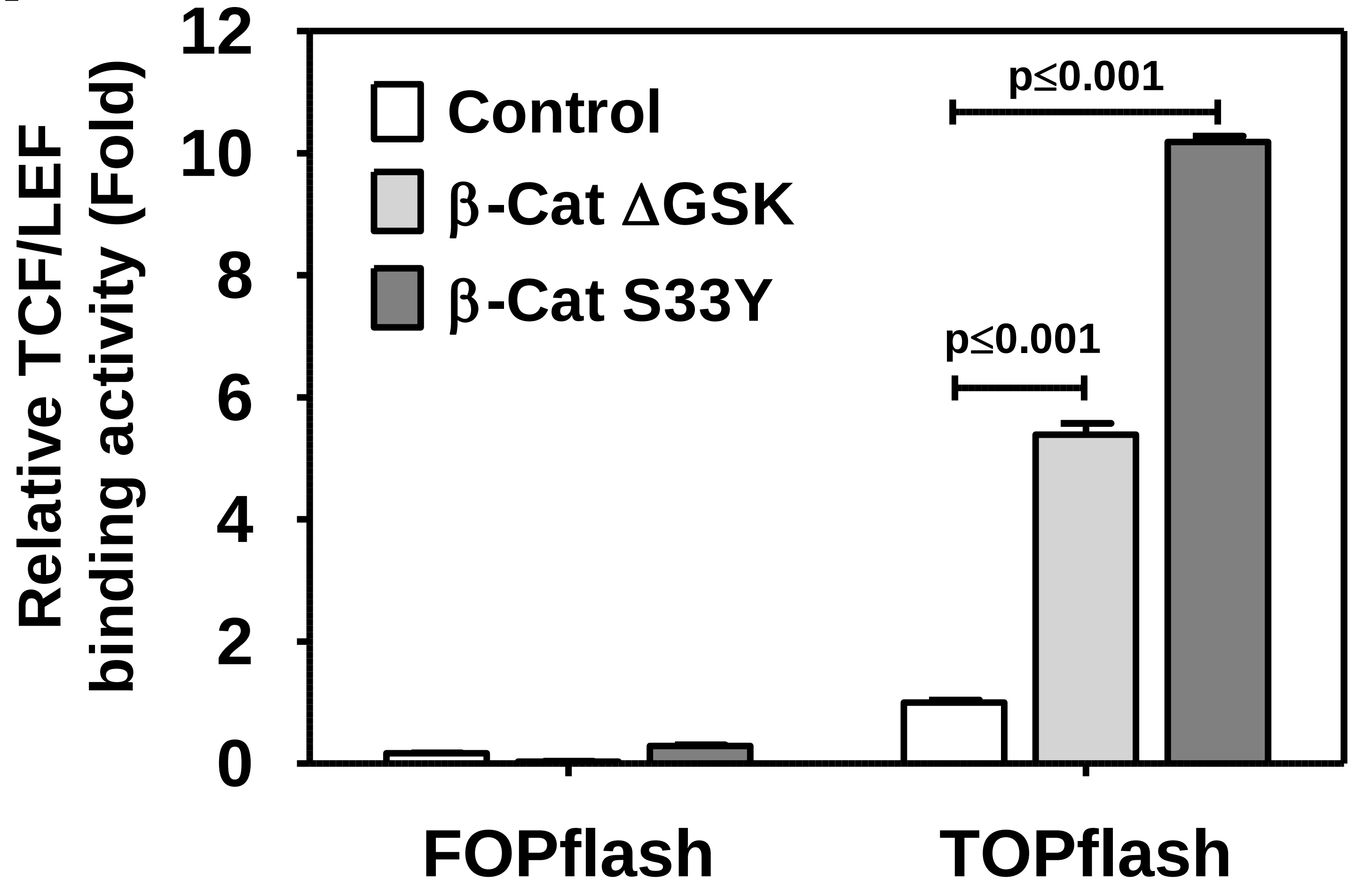

b

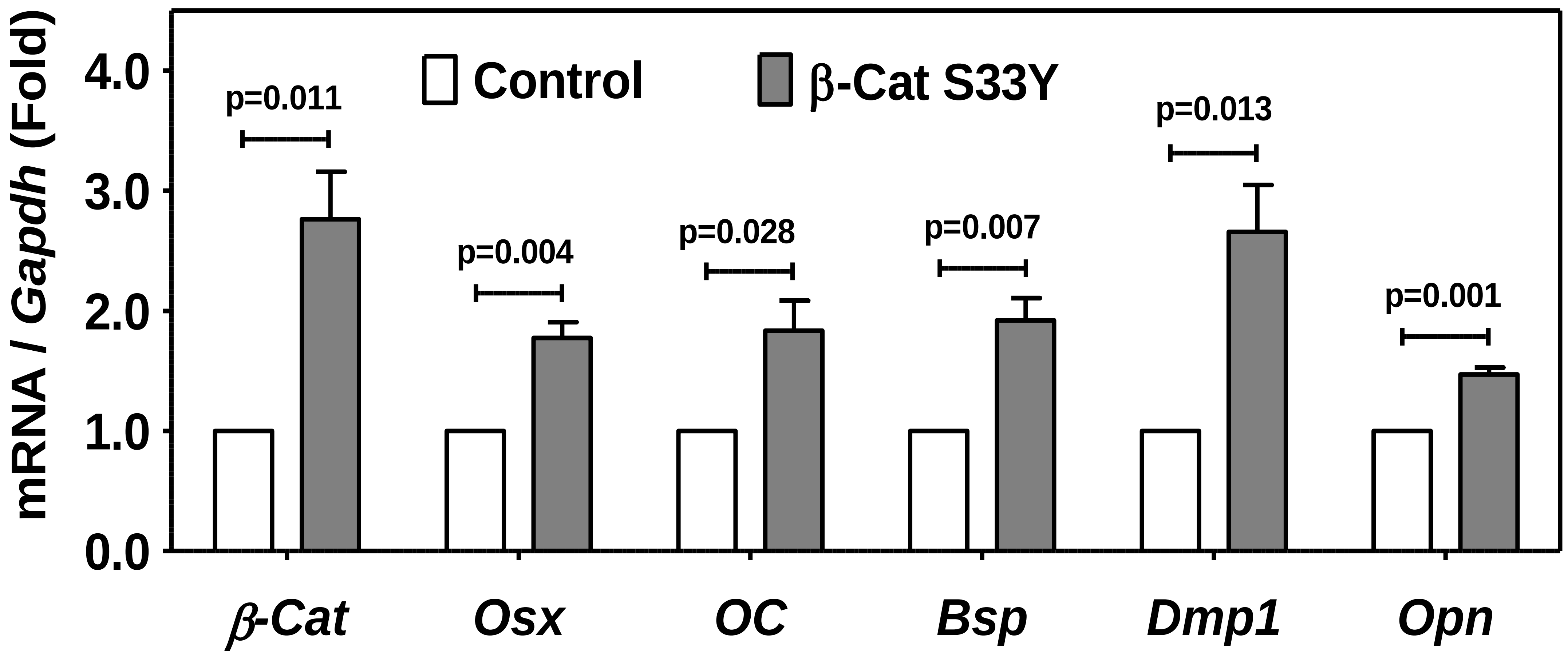

c

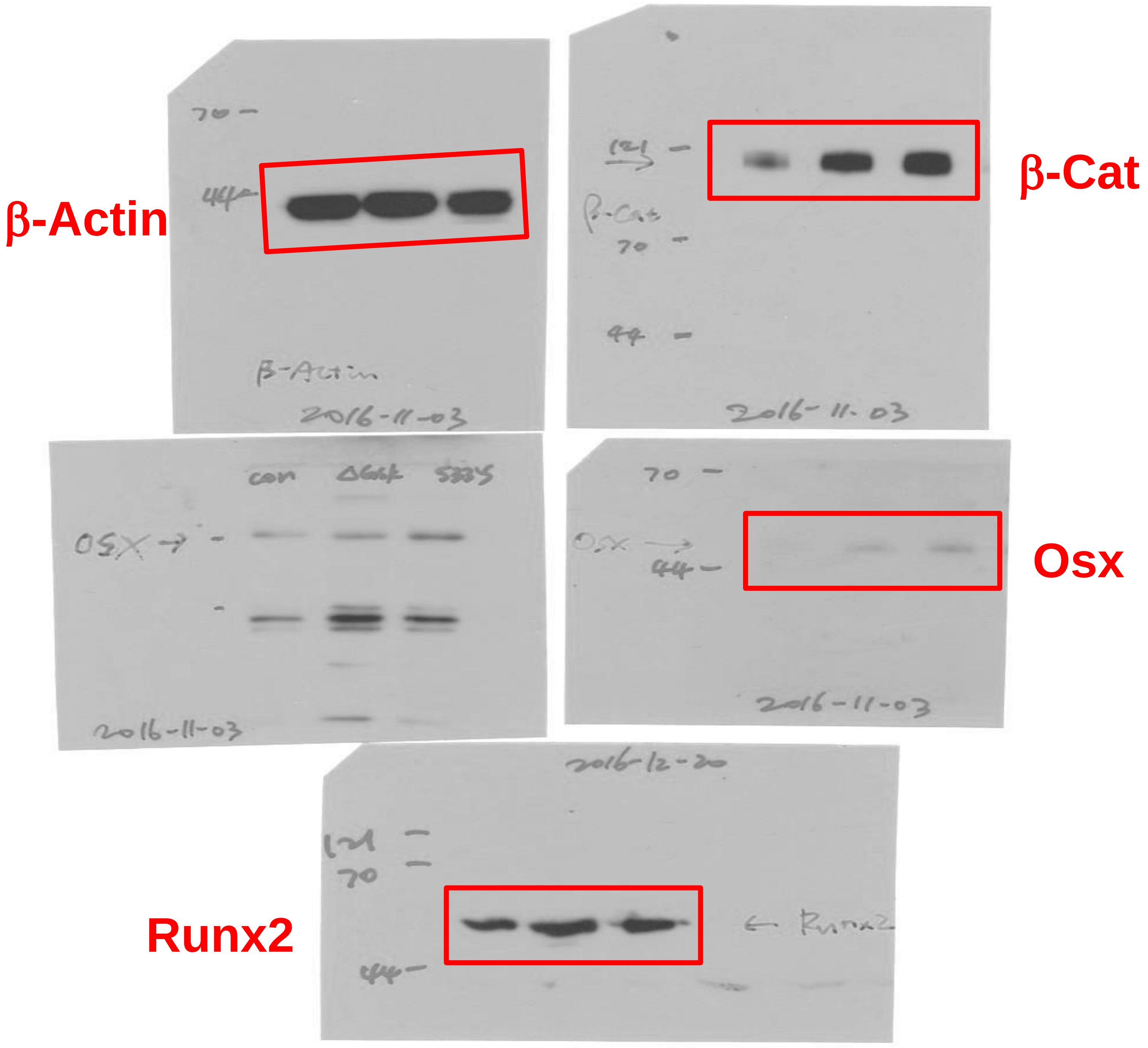

Supplementary Figure 3

a

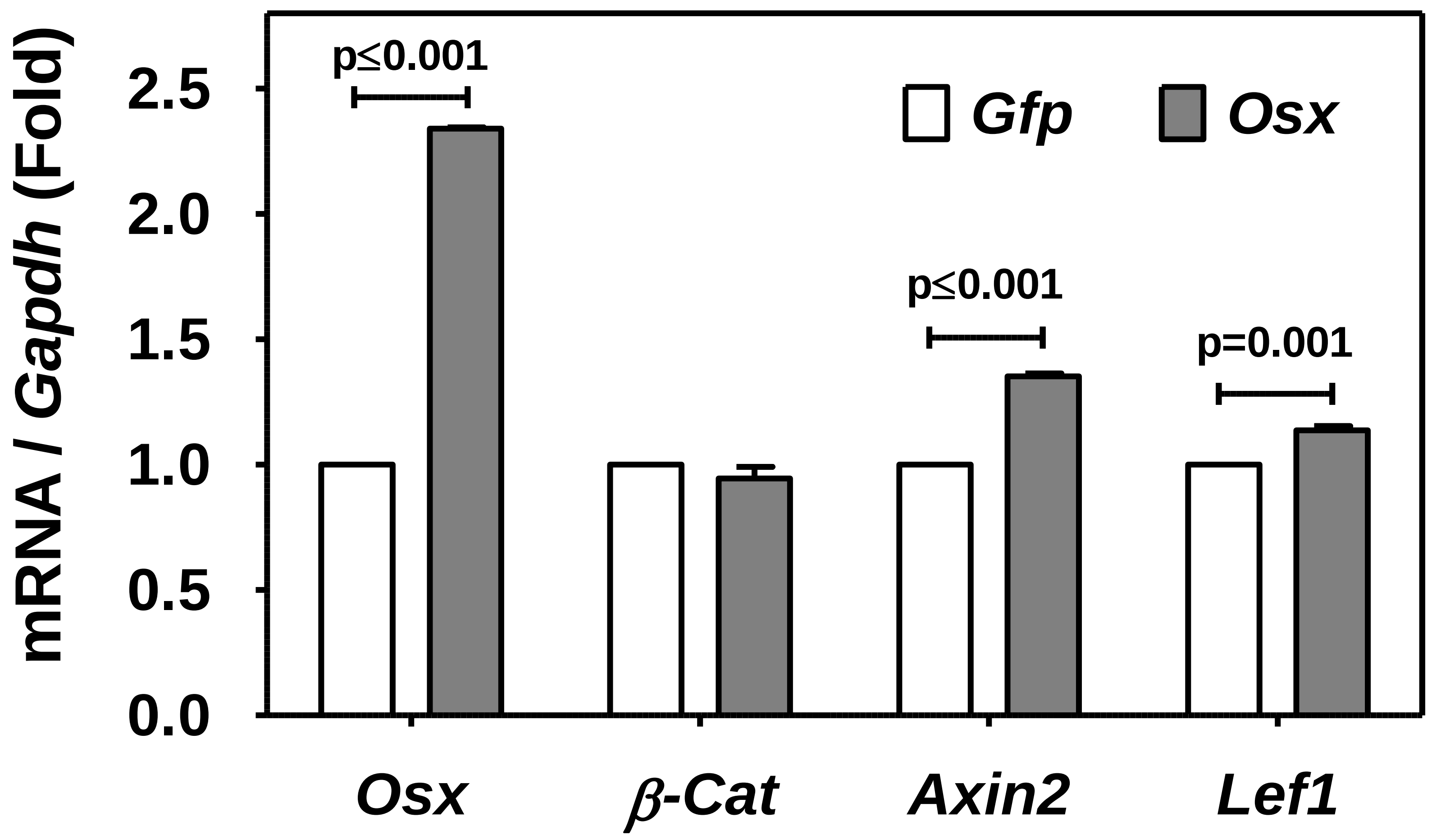

b

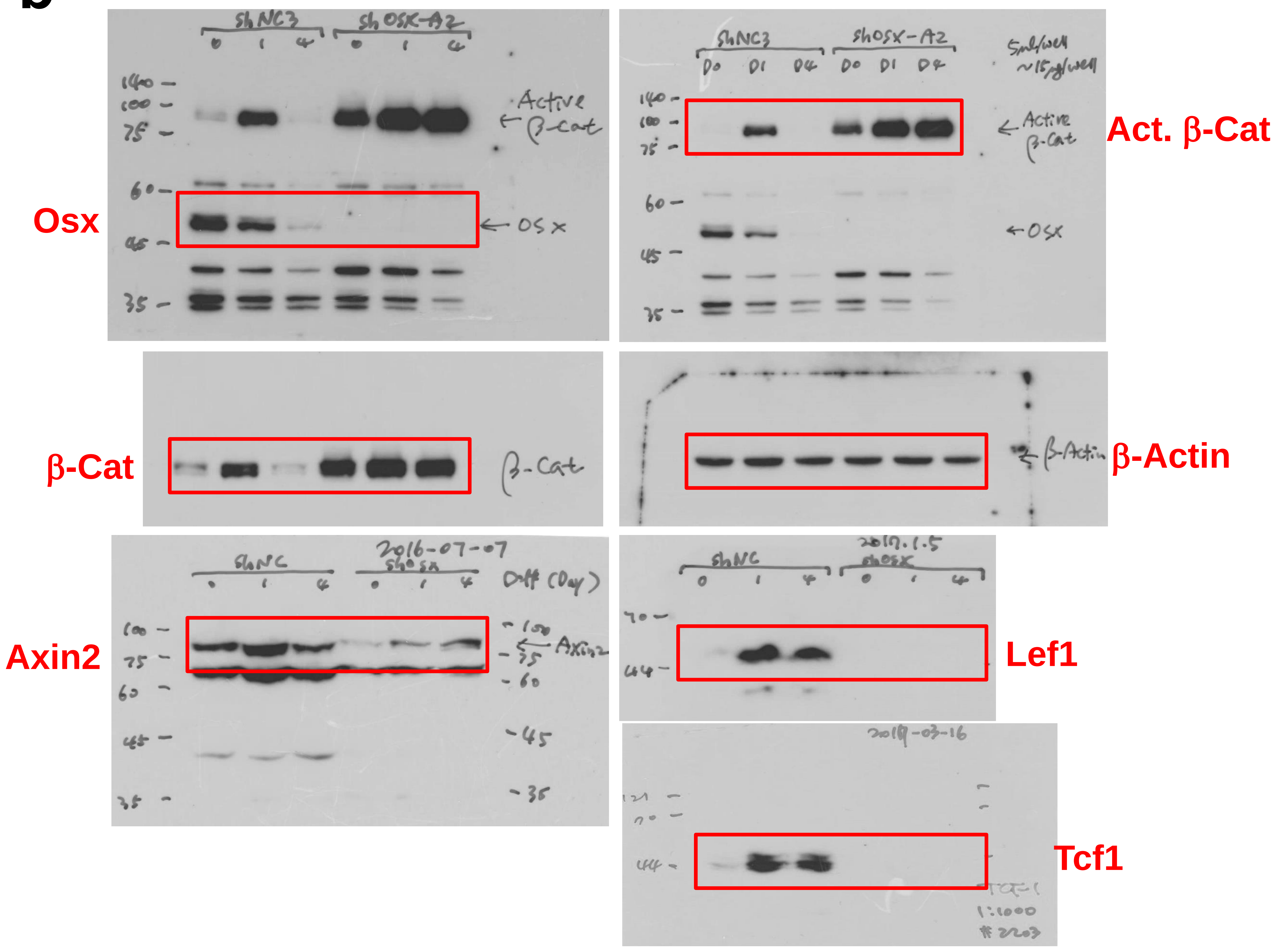

Supplementary Figure 4

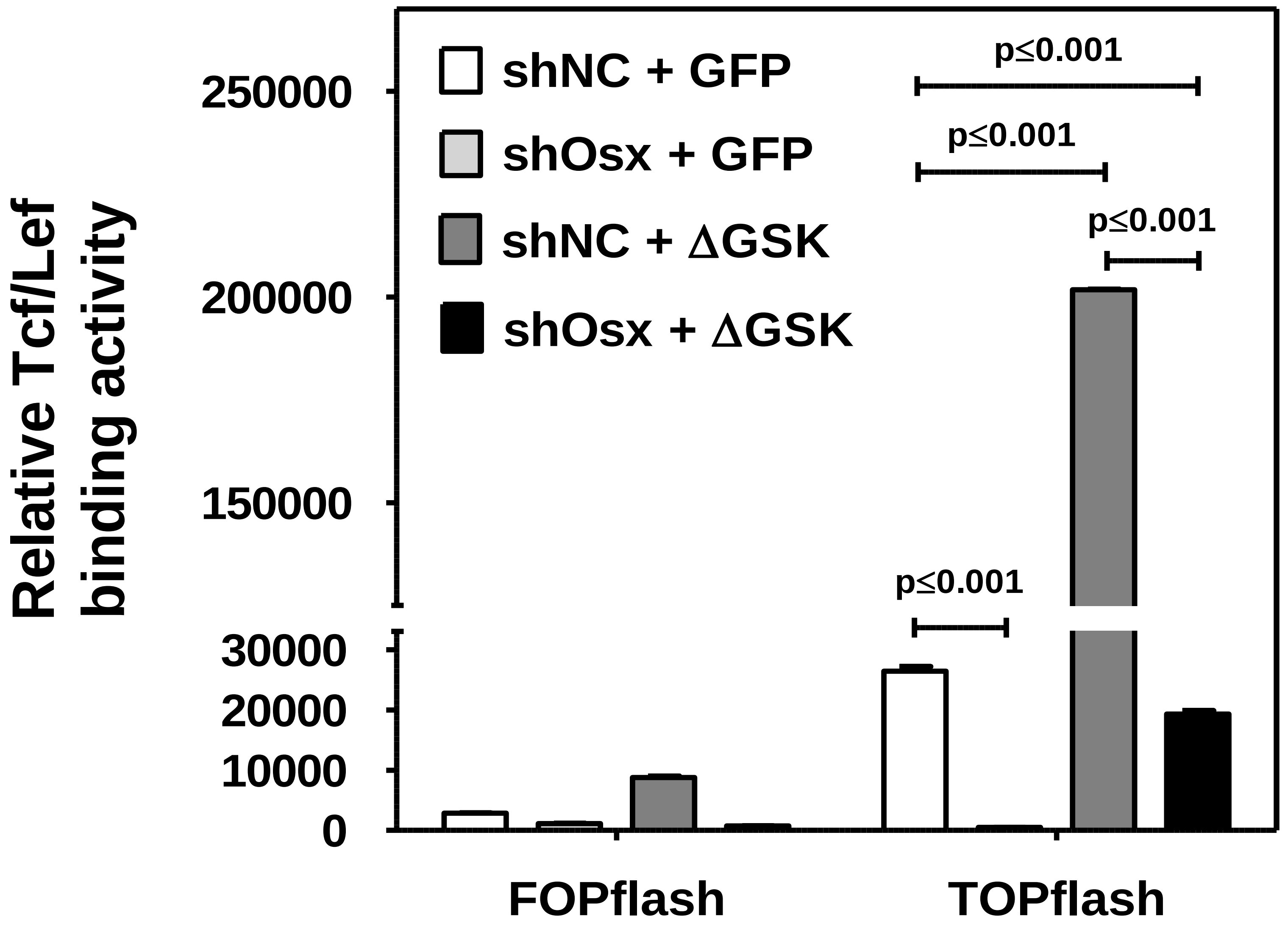

Supplementary Figure 5

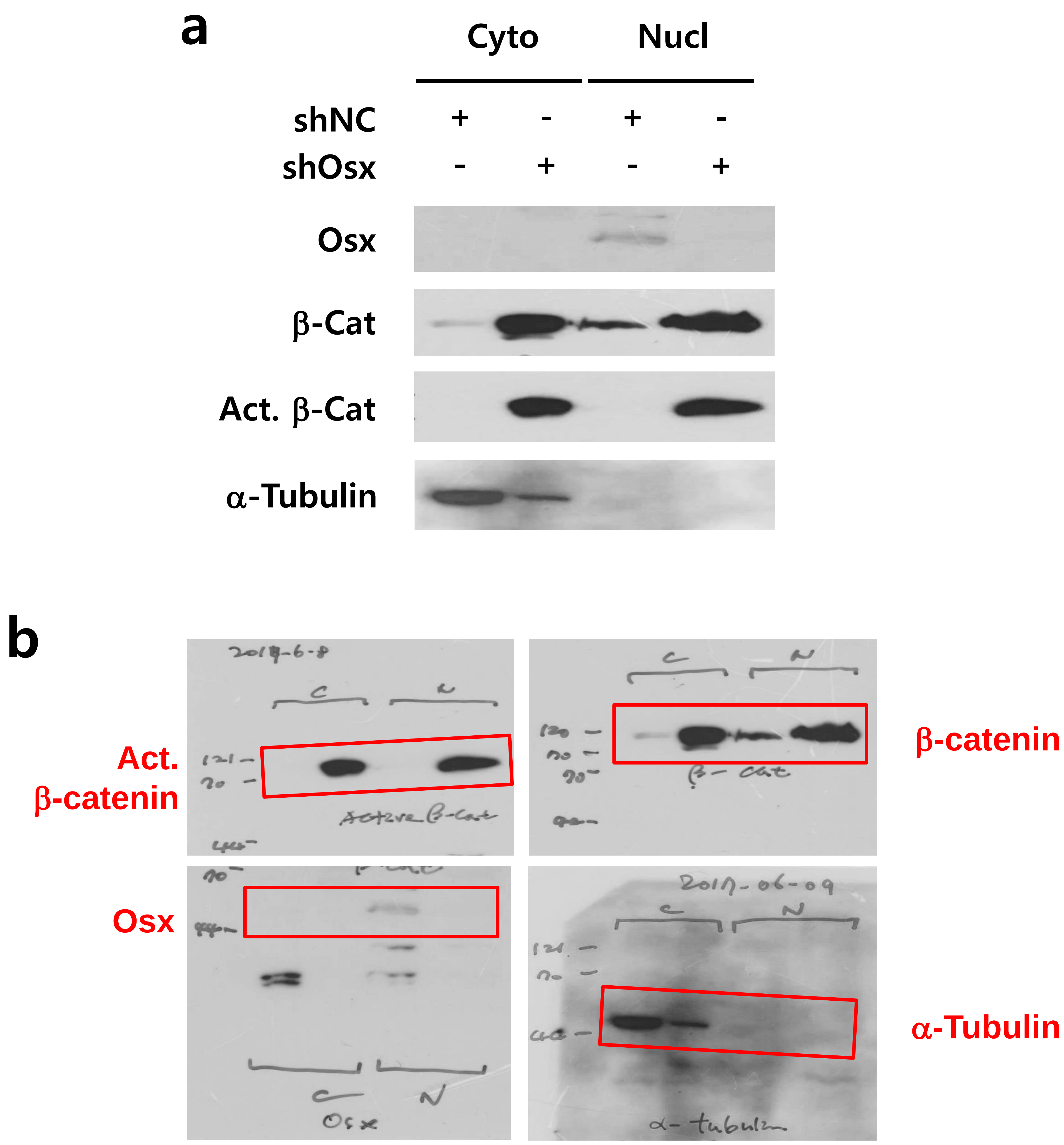

Supplementary Figure 6

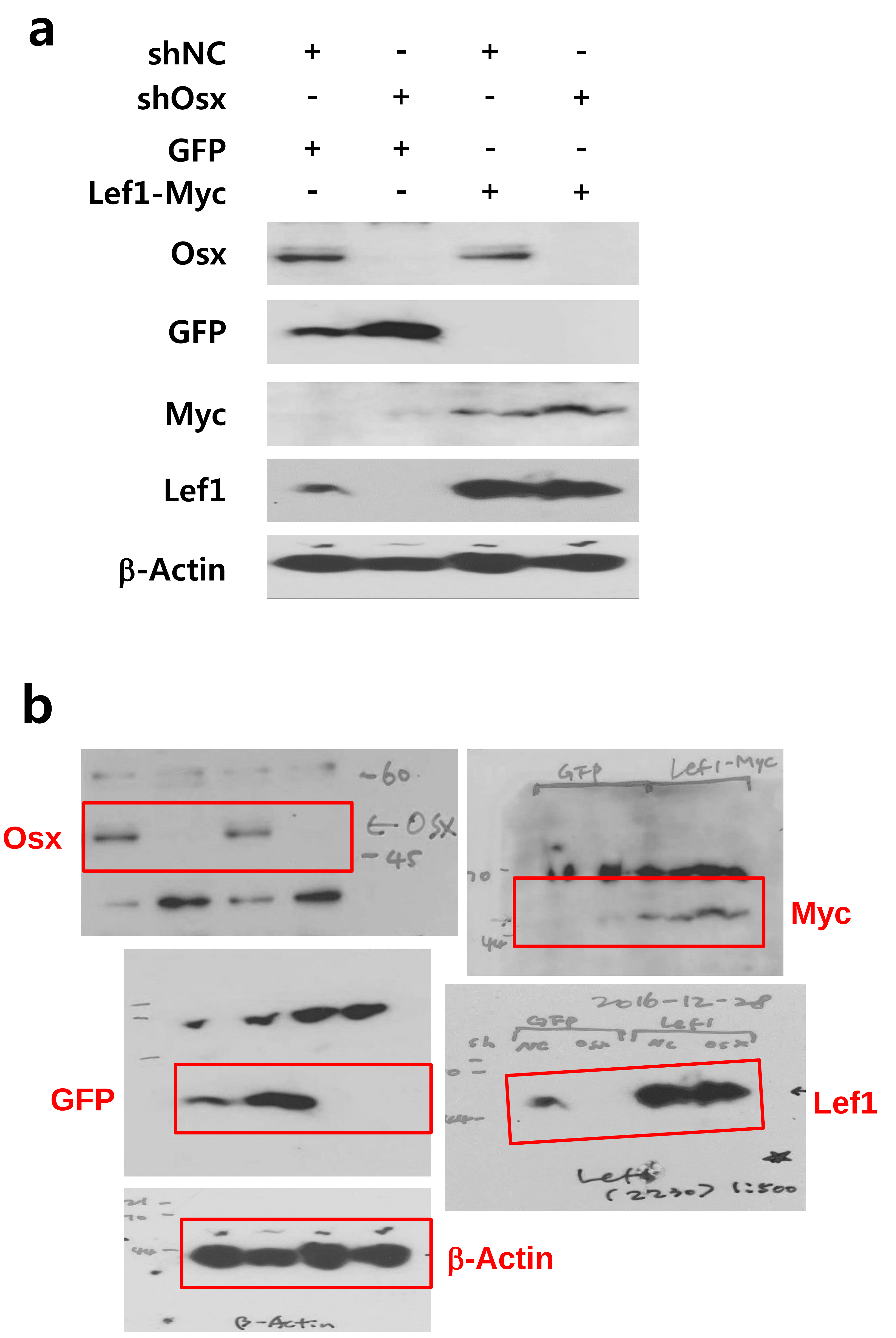

Supplementary Figure 7

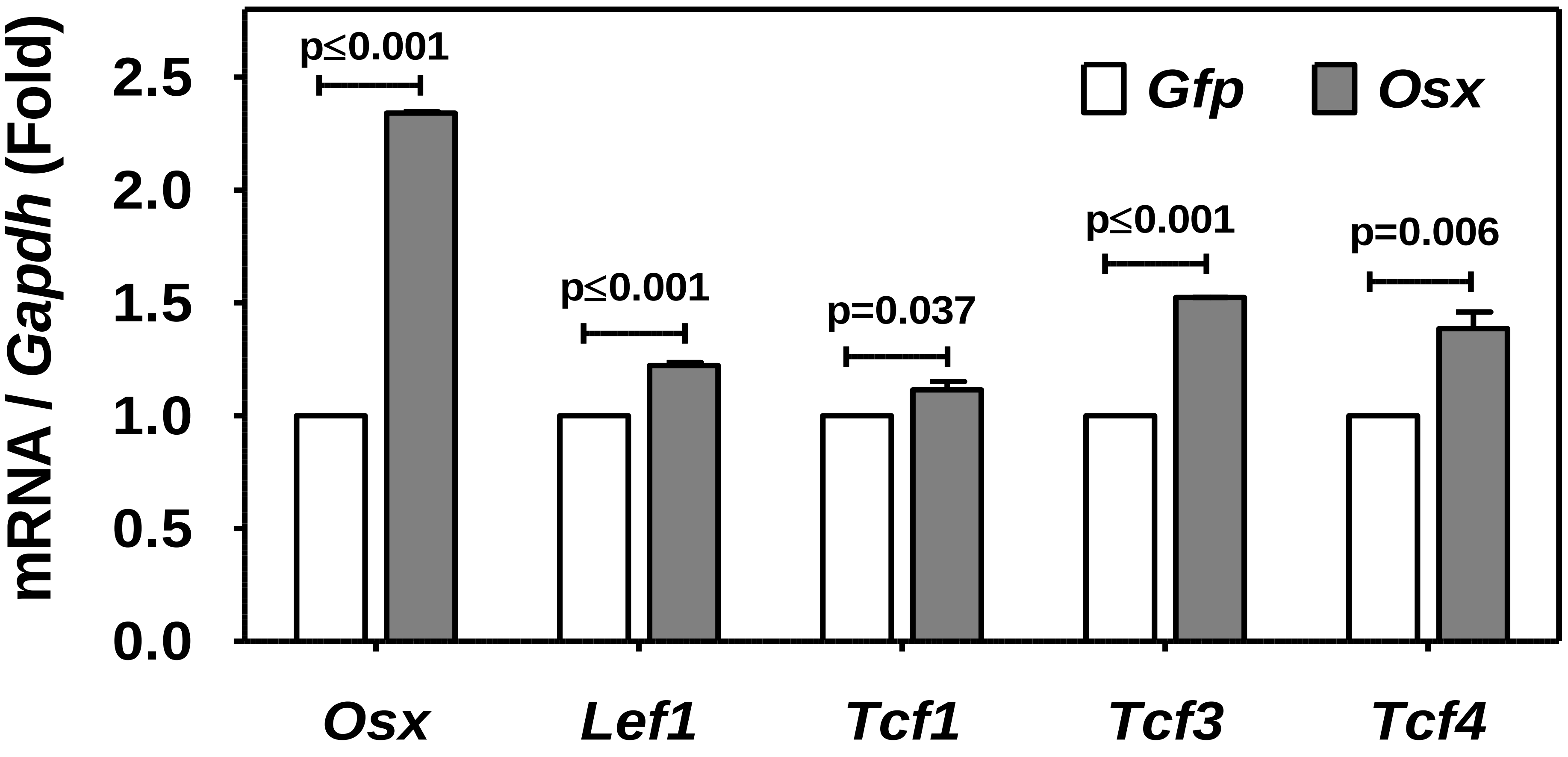

Supplement: Supplementary file 1 — Supplementary Information [file 41598_2017_8607_MOESM1_ESM.pdf]
